# Energy management and demand side management framework for nano-grid under various utility strategies and consumer’s preference

**DOI:** 10.1038/s41598-024-74509-y

**Published:** 2024-10-28

**Authors:** Youliana Bimen Elias, Mariem Y. Yousef, Abdalla Mohamed, A. A. Ali, Magdi A. Mosa

**Affiliations:** Electrical Power and Machines Engineering Department, Faculty of Engineering, Helwan University, Cairo, Egypt

**Keywords:** Nano-grid, Energy management, Demand side management, Load shifting, Consumer’s preference, Moth-flame optimization (MFO), Lagrange multiplier, Grid-outage, Grid pricing, Renewable utilization, Electrical and electronic engineering, Energy grids and networks, Renewable energy

## Abstract

This research proposes a day-ahead scheduling utilizing both demand side management (DSM), and Energy Management (EM) in a grid-tied nanogrid comprises of photovoltaic, battery, and diesel generator for optimizing the generation cost and the energy not supplied (at grid-outage). Wider terminology is introduced to combine both load controllability (considered in traditional DSM), and interval capability to accommodate additional loads defined as flexible, non-flexible, and semi-flexible intervals. Moreover, the user selection for EM or combined operation of EM with DSM at different degrees of interval flexibility is defined as user preference. In addition, three utility’s operations are considered denoted as fixed rate pricing (FRP), time-of-use (ToU) pricing, and FRP with grid-outage. Hence, the suggested framework utilizes the opportunities of generation diversity, the electricity pricing strategy, and the load flexibility. The obtained result show that, DSM with flexible intervals reduces the cost by 21.02%, 25.23%, and 18.15% for FRP, ToU, and FRP with grid-outage scenarios respectively. And cost reduction by 20.41%, 22.42%, and 17.81% for DSM with semi-flexible intervals and 16.24%, 21.15%, and 13.8% for DSM with non-flexible intervals. This cost reduction is associated with full utilization of renewable energy generation and reduction of the energy from/to battery which enhances its lifetime or reduces the required battery size during design stage for cost and provisions saving in flexible and semi-flexible intervals. A hybrid optimization technique of Moth-flame optimization algorithm, and Lagrange’s multiplier is proposed and confirms its effectiveness with detailed comparison with other techniques.

## Introduction

### Background

The residential sector plays a vital role in terms of its impact on overall power balance, stability, and efficient power management^1^. In addition, increasing penetration of renewable energy sources (RESs) into an existing distribution network is a driving force to develop a nano-grid (NG)^2,3^. This NG is a single-domain power system that may be connected to or disconnected from other power entities^4–6^ which is designed to feed a single house or small building^7,8^. Energy management system (EMS) optimizes the generation considering the opportunity in the generation and energy storage^3^. While involving the load in optimizing the NG operation is called demand side management (DSM). The DSM is a co-operative action by the customer and the utility to encourages the consumer to modify the consumption pattern and thus alter the configuration of the load curve^9,10^. This demand response program is classified into incentive based and time based. For incentive base technique, the utility provides incentive to the customer to adjust its consumption for technical-economic considerations. For time-based technique, the network power effectively is managed using electricity pricing such as Real Time Pricing (RTP)^3^, Time of Use (ToU), and Critical Peak Pricing (CPP)^9^. The DSM program not only enhances power grid security but also lowers operational costs^11^. To optimize the operation of the NG an efficient framework shall be applied, which will be able to deal with the grid’s strategies. Also, the framework shall be able to manage the load and the generation mix (renewable generation, local conventional generation such as diesel or biomass DGs, utility grid). The traditional DSM classifies the loads into shiftable (controllable) and non-shiftable (uncontrollable) without considering the interval capability to handle more or less loads. This paper introduces wider terminologies to combine both load controllability and interval capability to accommodate additional loads defined as flexible interval, non-flexible interval, and semi-flexible interval. Moreover, the user selection for EM or combined operation of EM with DSM at different degrees of interval flexibility is defined as user preference. Moreover, the user preferred operation strategy is an essential issue to provide applicable solutions. Furthermore, the framework shall be able to interact with the grid outage case which may result in shortage in generation. Additional challenges in implementing NG system such as nano-grids standard shortage, forecasting of renewable generation, absence of inertia such as PV, proper sizing of nano-grid components, … etc. These challenges require additional efforts to provide reliable, applicable, and interactive framework.

### Literature review

An overview of the DSM initiatives in the U.S., Australia, and UK with the aim to evaluate the potential of utilizing DSM as a solution in addressing the challenge of power electricity market has been presented in ref^12^. The operation mode and the benefits obtained by the DSM implementation which deals with number of challenges such as tariff regulation, energy transmission, distribution, and effective utilization of energy resources have been discussed in ref^13^. In ref.^8^, the DSM has been applied on a small scale grid-connected multi NGs consisting of Photovoltaic Panels (PV), Wind Turbines (WT), Diesel generator (DG) and battery energy storage system (BESS) in order to minimize the daily operating cost using manual load shifting. This manual load shifting reduces the generation cost, but it doesn’t provide the maximum utilization of load shifting technique. DSM has been utilized for peak load reduction through manual load shifting in ref.^14^, while effect of load shift on generation cost has not been taken into consideration. In ref.^15^, DSM analysis has been performed using two techniques peak clipping and load shifting techniques. Load clipping has been implemented during peak hours while this load clipping technique causes load curtailment. Load shifting has been implemented from peak to off peak for peak reduction only. In ref.^16^, load shifting DSM has been undertaken on three different grid connected sectors (residential, commercial, and industrial) with different consumption patterns and different quantities of fixed and movable loads in real-time electricity price (RTP) to achieve a reduction in peak loads and cost savings. In ref.^17^, DR strategies has been implemented for the three sectors with ToU and RTP strategies. However, grid outage has not been considered. In ref.^18^, A comparative performance study of six DR programs (time based and incentive based) has been conducted for microgrid sizing (planning) without considering the run time operation. In ref.^19^, an optimal demand-side flexible resources configuration scheme is obtained with high robustness against uncertainties of these resources. In ref.^20^, a novel approach for identifying potential customers using multi-criteria decision-making (MCDM) for DSM and net metering (NM) programs have been introduced for benefits of user and utility. In ref.^21^, load shifting based DSM has been implemented for grid-connected and grid islanding microgrid at different level of shiftable load using time of use pricing (ToU). While grid interruption, user preference, energy storage have not been considered. In ref.^22^, the DSM has been used to reduce the peak demand and to provide substantial savings in energy bill. In ref.^23^, a two-stage EMS considering DSM utilizing ToU and load curtailment to manage power generation and reduce operational costs considering uncertainty in generation and price has been presented. In ref.^24^, DSM has been used to minimize the peak load and peak time stress on grid for ToU program, but the Impact of DGs and storage devices has not been considered. In ref.^25^, optimal energy scheduling of an energy hub system considering the participation of DSM is implemented in storage based residential buildings at day-ahead. In ref.^26,27^, user-centric optimization strategy offers NG users a set of solutions for their preferred load shifting based on their comfort levels and desired for cost reductions. Linear programming and mixed-integer linear programming in ref.^28^ solve the dispatch problem of a nano-grid with different resources in five various operating modes used to operate the NG depending upon the strategies of grid connectivity, the direction of power flow and the availability of various distributed resources. A demand response scheme is employed in ref.^29^ to formulate the performance index of the energy management system on a developed NG model to maximize energy from the solar panel and battery storage while minimizing the power received from the utility grid. Reference^30^ proposes an energy-efficient storage capacity approach, that combines multiple load power flow assignment with a load-shifting algorithm to minimize energy loss and determine the optimal energy storage capacity. Reference^31^ presents a real-time energy management system, load shifting and tuning for an off-grid smart house that uses a fuzzy logic controller to manage controlled loads in order to efficiently use excess renewable energy and increase the demand supplied by these resources while maintaining the user’s comfort level. An energy management with demand response program strategy is proposed in ref.^32^, which uses the variations in the grid tariff to charge the BESS when the tariff is low and reduces the peak demand, which reduces the levelized cost of energy.

On the other hand, EM and DSM are non-linear and complex problems which is hard to be optimized^33,34^. Deterministic techniques, such as mixed integer and non-linear programming using Branch and Reduce Optimization Navigator (BARON) for EM of a microgrid comprises conventional generation, modern generation and energy storage system has been applied in ref^33^. Metaheuristic techniques have proven its efficiency to hand complex, discontinues, and non-convex problems such as Ant Lion Optimization^24^, PSO and the Strawberry optimization technique (SBY)^17^, Aquila optimizer^8^, Cheetah Optimizer Algorithm (COA)^11^, Shuffled frog leaping algorithm (SFLA)^25^, a hybrid approach joints the implementation of the Wingsuit Flying Search Algorithm (WFSA) and Artificial Cell Swarm Optimization (ACSO)^35^, and a Genetic Algorithm (GA) with Simulated Annealing optimization Algorithm (SAA) were proposed in ref^36^. In ref^34^, a hybrid method of the PSO and sequential quadratic programming (SQP). In this article, a hybrid Moth-flame optimization (MFO) technique and Lagrange multiplier is proposed to adjust the demand profile and manage the available generation resources to optimize the generation cost and minimize the energy not supplied for a NG comprises of photovoltaic system, diesel generator, and battery (Table 1).

**Table 1:** Comparison of literature works related to MG/NG energy management (EM) and demand side management (DSM).

Year	Ref. no.	System	Grid scenarios			Optimizer			Objective function				EM	DSM	User preference
			Grid-connected	Grid islanded	Grid outage	Det.	Meta.	Hybrid	Operation cost	User comfort	E_*N*.S_	REC			
2023	^8^	MNG	**√**	**–**	**–**	**–**	**√**	**–**	**√**	**–**	**–**	**–**	**√**	**√**	**–**
2020	^14^	NG	**√**	**–**	**–**	**–**	**–**	**–**	**√**	**–**	**–**	**–**	**–**	**√**	**–**
2022	^16^	MMG	**√**	**–**	**–**	**–**	**√**	**–**	**√**	**–**	**–**	**–**	**–**	**√**	**–**
2021	^17^	MG	**√**	**–**	**–**	**–**	**√**	**–**	**√**	**–**	**–**	**–**	**–**	**√**	**–**
2023	^18^	MG	**√**	**–**	**–**	**–**	**√**	**–**	**√**	**–**	**–**	**–**	**√**	**√**	**–**
2023	^21^	MG	**√**	**√**	**–**	**–**	**√**	**–**	**√**	**–**	**–**	**–**	**√**	**√**	**–**
2019	^23^	MG	**√**	**–**	**–**	**–**	**–**	**√**	**√**	**–**	**–**	**–**	**√**	**√**	**–**
2022	^35^	MG	**√**	**–**	**–**	**–**	**√**	**–**	**√**	**–**	**–**	**–**	**–**	**√**	**–**
2023	^49^	MG	**√**	**–**	**–**	**–**	**–**	**√**	**√**	**–**	**–**	**–**	**√**	**–**	**–**
2022	^50^	MG	**√**	**–**	**–**	**√**	**–**	**–**	**√**	**–**	**–**	**–**	**√**	**–**	**–**
2023	^51^	MG	**√**	**–**	**–**	**√**	**–**	**–**	**√**	**–**	**–**	**–**	**√**	**√**	**–**
2019	^52^	MG	**√**	**–**	**–**	**√**	**–**	**–**	**√**	**√**	**–**	**–**	**√**	**–**	**–**
2023	^53^	MG	**√**	**–**	**–**	**–**	**–**	**√**	**√**	**√**	**–**	**–**	**–**	**√**	**–**
2021	^54^	MG	**√**	**–**	**–**	**–**	**–**	**√**	**√**	**√**	**–**	**–**	**–**	**√**	**–**
Proposed		NG	**√**	**–**	**√**	**–**	**–**	**√**	**√**	**–**	**√**	**√**	**√**	**√**	**√**

NG: Nano-grid, MG: Micro-grid, MMG: Multi Micro-grid, MNG: Multi Nano-grid.

User Comfort means the sensitivity towards the load shifting along the day as ref^52^ or the unshifted hours for each load to all the operating hours as ref^53^ and ref^54^. But user preference means that the user can select between EMS and DSM with different degrees of interval flexibility as will be discussed later.

### Research gaps

Based on the research overview the following research gaps are proposed in the field of grid-connected Nano-grid operation:

1. In literature, several researchers assume the user’s consumption is shiftable and controllable in all intervals along the day without considering the user preference. Therefore, user reference shall be involved in the operation framework. For example, the user may prefer to keep the load fixed during certain intervals (without addition/reduction the load), or the load is not shiftable while its associated interval can receive additional loads from other intervals (permissible additional loads only).
2. In literature two cases have been investigated known as grid connected NG (the NG is permanently connected to the utility grid), or grid islanded (the NG is permanently islanded from the utility grid). While grid outage for some operating intervals in grid-connected system have not been taken into consideration. During the grid-outage, the available generation (of the remaining units within the nano-grid) may be insufficient to supply the total load. As a result, the amount of the available energy and load pattern shall be optimized not only to optimize the operation cost but also to minimize the energy not supplied.
3. Several research optimize the system using deterministic techniques only or metaheuristic techniques only. The deterministic techniques are suffering from mathematical modeling complexity. On the other hand, metaheuristic techniques are suffering from randomness which may lead to trapping in a local minimum. Hence, a hybrid optimization technique may provide better performance. In addition, Numerous of research provide modified metaheuristic algorithms for solving EM or DSM problems ignoring the algorithm ability to achieve the optimal value over several trials. Therefore, the stability of modified algorithms to provide the optimal solution over several runs shall be measured.
4. Several DSM systems are based on manual load shifting such as in refs.^8,14,15^, which do not ensure optimal operation of the nano-grid. Hence, the NG management framework which adjusts the load pattern in an optimal manner utilizing other opportunities such as the diverse of energy resources and grid pricing strategies needs investigation.

### Contribution

This paper proposes a framework that comprises a new formulation for EMS and DSM for different utility scenarios (Fixed pricing, and ToU). The problem formulation considers the user’s preference and classifies the operation intervals into flexible, non-flexible and semi-flexible intervals. In flexible intervals, the demand can be increased or decreased to optimize the objective function. The demand can be decreased means the load is controllable, and shiftable and can be shifted to other intervals and the demand load value decrease below its base value, while the demand can be increased means the interval can receive additional loads from the other intervals and increase the new load demand value above its predefined value. Non-flexible intervals, the demand cannot change from its predefined value, hence this interval doesn’t participate in the DSM directly. Semi-flexible intervals, the load cannot decrease below its base value (uncontrollable load) while the interval can receive additional load from the other intervals to enhance the outcomes of the DSM. Eventually, managed grid disconnection (at predefined intervals along the day due to load shedding program by the utility) is considered which requires to manage the load and available generation resources to minimize the expected energy not supplied as well as generation cost. The proposed framework utilizes electricity pricing mechanism (Fixed pricing, and ToU), the different generation technologies (PV as low operation cost, the battery as flexible unit to charge/discharge, utility, and diesel as different generation cost), and the load shifting as DSM to provide optimal operation of the NG. To enhance, the conversion of the optimization technique a hybrid metaheuristic based MFO, and deterministic Lagrange technique is proposed. The main contributions of this paper can be summarized as:

1. Introducing an efficient framework considering the opportunities of EM (which utilizes the presence of different generation technologies and the grid pricing strategy) and DSM (which utilizes the load flexibility) for total generation cost minimization. Moreover, employing DSM technique and the available generation energy storage resources to optimize both the generation cost and the energy not supplied due to generation shortage at grid outage to address the possibility of insufficient generation.
2. Proposing a classification of loading intervals denoted as flexible interval, non-flexible interval, and semi-flexible interval. These intervals’ notation combines the load controllability and the interval capability to receive additional loads from the reset intervals. These notations are different from the conventional load classifications which consider the load controllability without considering the limitations introduced by the user on each interval. The user selection between EM or combined operation of EM with DSM at different degree of intervals flexibility is defined as user preference.
3. The suggested framework is capable of handling different utility pricing strategies (such as fixed rate pricing and time of use pricing), and different user preferences (such as EM and DSM with flexible intervals, semi flexible and non-flexible intervals), and generation outage intervals (utility managed grid outage). These features make the proposed framework adaptable for practical applications.
4. Demonstrate a hybrid optimization technique which combines the merits of metaheuristic technique (MFO) and deterministic technique (Lagrange) to provide global solution and enhance the robustness of the problem solution. The solution robustness is verified through several independent trials. The performance of the suggested technique is promising compared to particle swarm optimization (PSO) and the MFO optimization techniques.

### Paper organization

The rest of the paper is structured as follows: section “Grid connected nano-grid” presents the structure of the grid-connected NG under investigation. Section “Proposed management framework of the nano-grid” elaborates the proposed EMS and the DSM of the NG considering different customers’ preferences. The suggested optimization technique involving MFO and Lagrange is summarized in section “Hybrid MFO and Lagrange”. The simulation results of different operation modes are analyzed and investigated in section “Study cases”. Section “Generation cost and Battery participation during different scenarios” investigates the battery and generation cost during different operation scenarios. Comparison between the proposed hybrid technique (MFO with Lagrange), MFO only, and PSO is introduced in section  “Evaluation of convergence of the system used algorithms”. Finally, the conclusion drawn from the presented work and the future research are described in section  “Conclusion and future research”.

## Grid connected nano-grid

**Fig. 1:**
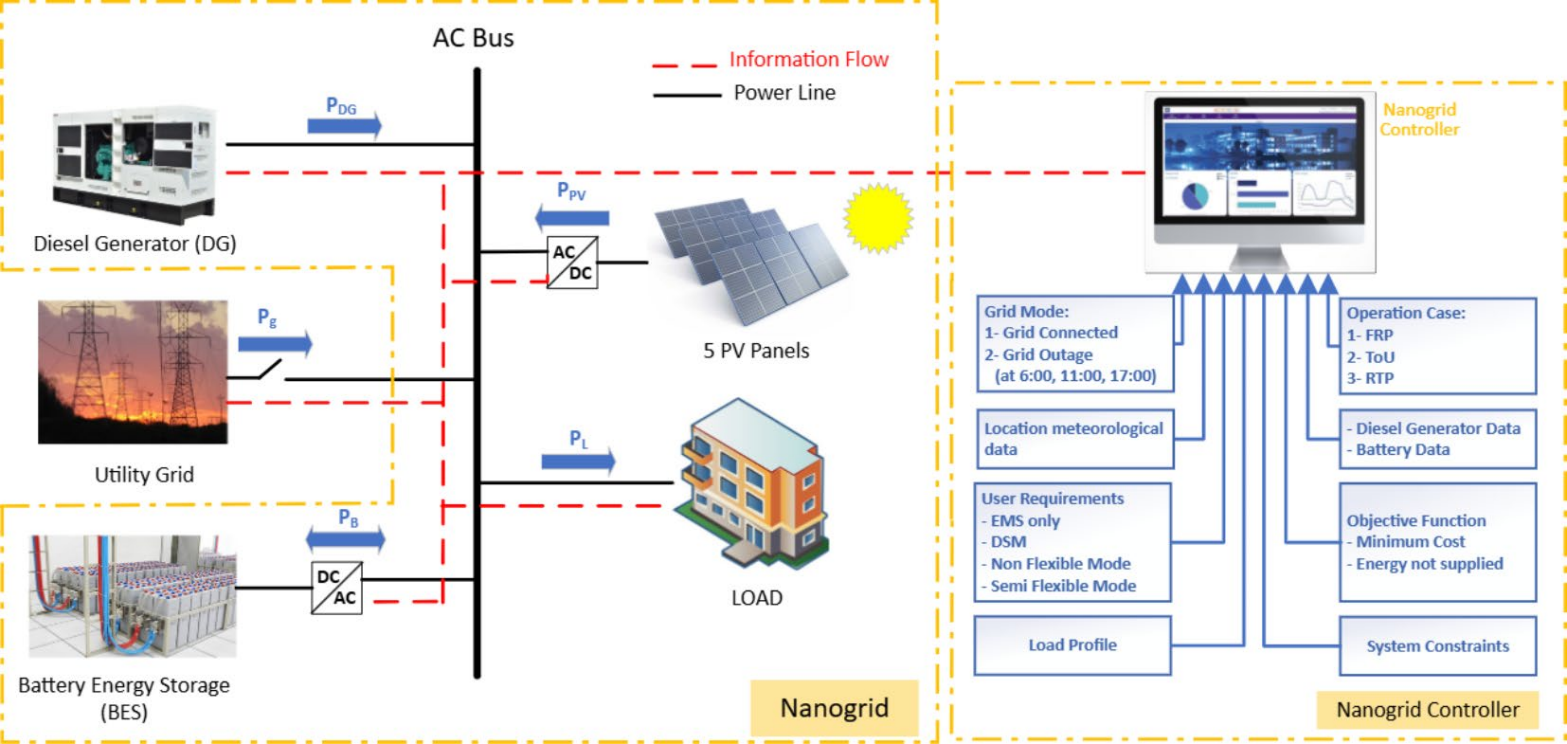
Proposed nano-grid.

The system under study consists of grid-tied NG tied as shown in Fig. 1. The nanogrid has the same architecture as in ref^8^ (grid connected nanogrid comprises PV, battery, Diesel, and load) with the same PV profile, almost the same load profile, and slight modification in the diesel and battery ratings. The PV rating is 20 kW, the diesel rating is 12 kW, and the battery capacity of 24 kWh. Each source in the NGs has its own operating manner. For example, the battery operates in charging, discharging or idle mode, the PV system works in either maximum power point tracking or limiting power mode, the DG operates with its power limits according to NG management system, and the utility grid delivers power to the NG as per the NG management system. The proposed NG management controller measures the system variables in day-ahead after that determines the optimal operating mode and power of each source as well as the optimal system demand to optimizes the system objective function. The NG management system shall maintain the system variables within acceptable and safe limits as given in Table 2.

**Table 2:** Nano-grid data.

	PV	DG	Battery	Grid (During grid connected)	Grid (During grid outage)
Maximum power (kW)	20	12	6	50	0
Minimum power (kW)	0	2.4 or stop	− 6	0	0
maximum SOC (%)	–	–	1	–	–
Minimum SOC (%)	–	–	0.3	–	–
Energy (kWh)	–	–	24	–	–

## Proposed management framework of the nano-grid

To maximize the benefits of the NG, an efficient management system involving both EM and DSM is proposed. The EMS dispatches the power generation between resources to maintain the balance between generation and demand, while in demand side management, part of the load is shifted to achieve several benefits such as economic operation (minimum cost) and technical issues such as minimizing the amount of energy not supplied during generation shortage.

### Objective function

The system objective function depends on the operation condition. For grid connected operation the utility grid covers the local generation shortage hence, the management system minimizes the daily generation cost as given by Eq. (1)^8^. Where $\:{{C}_{g}\left(t\right),\:{C}_{dg}\left(t\right),\:and\:C}_{B}\left(t\right)$ are the cost of energy supplied from the grid, the cost of energy supplied from the diesel and the cost of energy supplied from the battery respectively.

1 $$\:\text{min}{F}_{1}\:=\text{min}\sum\:_{t=1}^{24}\left({{C}_{g}\left(t\right)+{C}_{dg}\left(t\right)+C}_{B}\left(t\right)\right)$$

During grid outage, without DSM, the remaining generation sources are the PV, DG, and battery, the available power capacity may be not sufficient to keep balance, hence the NG management system shall be designed to optimize the operation cost and minimize the load curtailment. The updated objective function is given in Eq. (2). Where the first term is the generation cost, and the second term is the power not supplied (mismatch between generation and load). $\:{W}_{1}$ and $\:{W}_{2}$ are weighting factors. $\:{P}_{g}\left(t\right),\:{P}_{dg}\left(t\right),\:{P}_{Apv}\left(t\right)$ are the power delivered from the grid, the diesel generator, PV system, $\:{P}_{Lo}\left(\text{t}\right),\:{P}_{B}\left(\text{t}\right)$ the delived power to the load and battery. With DSM, the energy not supplied shall also be minimized as given in Eq. (3), the only difference is that, without DSM the objective function considers original load ($\:{P}_{Lo}\left(\text{t}\right)$), while with DSM the objective function considers the modified load ($\:{P}_{L}\left(\text{t}\right)$). During intervals of grid outage $\:{P}_{g}\left(t\right)=0\:and\:{C}_{g}\left(t\right)=0\:$

2 $$\:\text{min}{F}_{2}\:=\sum\:_{t=1}^{24}\left[{W}_{1}\left({{C}_{g}\left(t\right)+{C}_{dg}\left(t\right)+C}_{B}\left(t\right)\right)+{W}_{2}\left(\:{P}_{g}\left(t\right)+{P}_{dg}\left(t\right)+{P}_{Apv}\left(t\right)-{P}_{Lo}\left(\text{t}\right)-{P}_{B}\left(\text{t}\right)\right)\right]$$

3 $$\:\text{min}{F}_{3}\:=\sum\:_{t=1}^{24}\left[{W}_{1}\left({{C}_{g}\left(t\right)+{C}_{dg}\left(t\right)+C}_{B}\left(t\right)\right)+{W}_{2}\left(\:{P}_{g}\left(t\right)+{P}_{dg}\left(t\right)+{P}_{Apv}\left(t\right)-{P}_{L}\left(\text{t}\right)-{P}_{B}\left(\text{t}\right)\right)\right]$$

The fuel cost of diesel generator is given by Eq. (4)^40^, where $$\:a,b$$ and $$\:c$$ are generator cost coefficients which can be obtained from manufacture data and fuel prices^37^. For the selected DG, the factors $$\:a=0.0111$$ $/kW^2^,$$\:\:b=0.0813$$ $/kW, and $$\:c=0.86$$ $.

4 $$\:{C}_{dg}\left(t\right)\:={a\left({P}_{dg}\left(t\right)\right)}^{2}+{bP}_{dg}\left(t\right)+c$$

The electricity cost from the grid is given by Eq. (5)^23^, where $\:{\rho\:}_{g}\left(t\right)$ is the grid tariff.

5 $$\:{C}_{g}\left(t\right)={\rho\:}_{g}\left(t\right){P}_{g}\left(t\right)$$

The cost of battery is given by Eq. (6). Where: $\:{\rho\:}_{B}$ is the battery cost coefficient. $\:\:\:{\rho\:}_{B}=0.0982\:\text{\$}/kWh$ which is calculated using method in ref^38^.

6 $$\:{C}_{B}\left(t\right)={\rho\:}_{B}{P}_{B}\left(t\right)$$

### Energy management constraints

EM optimally dispatches the power among generation units without violating the system and units’ operation constraints.

#### Power balance constraint

The balance between generation and demand must be maintained as shown in Eq. (7)^40^. The demand is the total load and charging power of the battery, while the generation is the grid power, diesel power and PV power.

7 $$\:{P}_{Lo}\left(t\right)+{P}_{B}\left(\text{t}\right)\:={P}_{g}\left(t\right)+{P}_{dg}\left(t\right)+{P}_{Apv}\left(t\right)\:\forall\:t\in\:\left\{\text{1,2},3,\dots\:,24\right\}$$

**Fig. 2:**
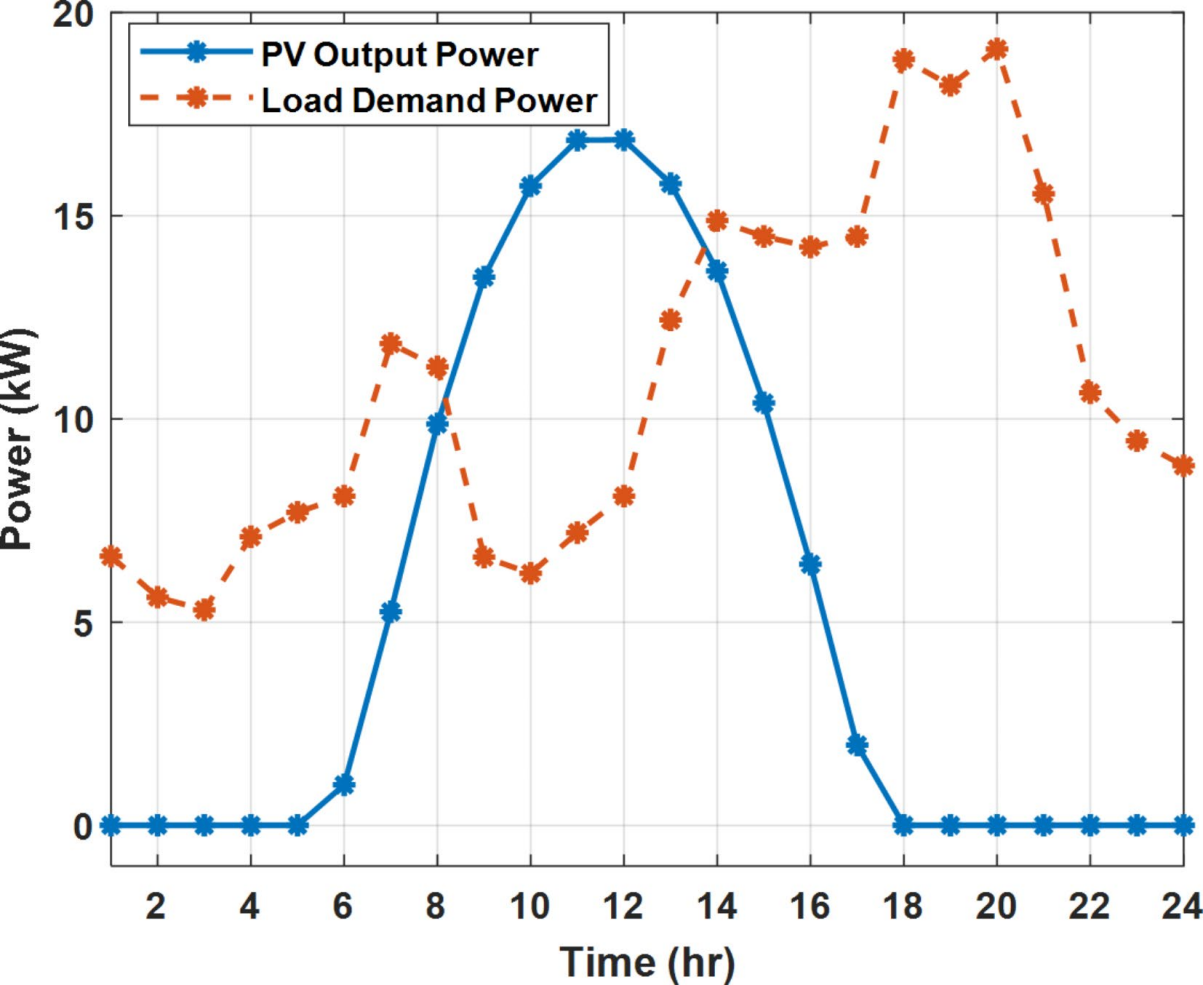
The predicted PV available power and load profile for 24 h.

**Fig. 3:**
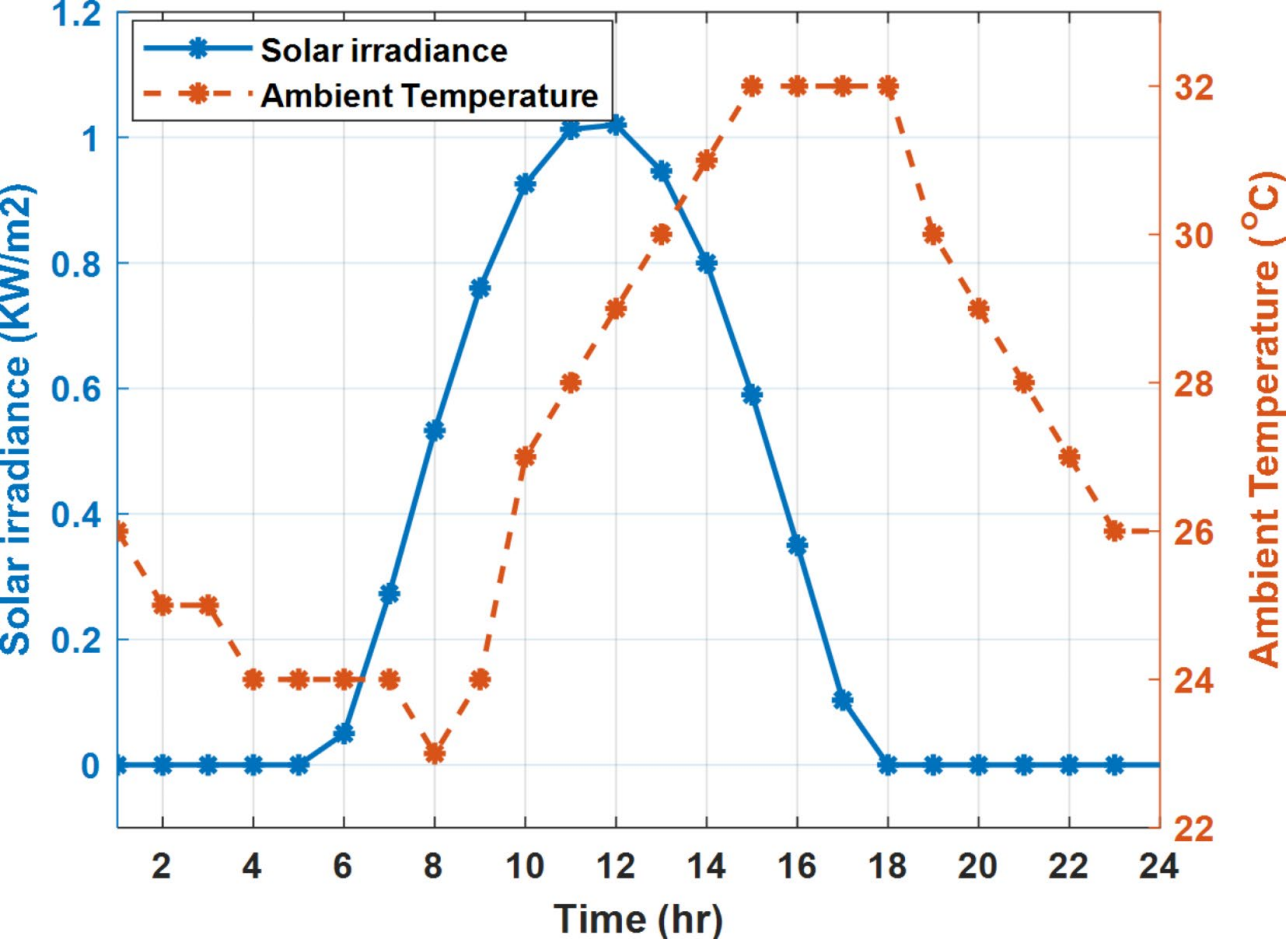
The predicted solar irradiance and ambient temperature for 24 h.

The daily load profile without DSM ($\:{P}_{Lo}\left(t\right)$) is shown in Fig. 2^8^. The available power of the PV system depends on the climatic conditions. The predicted solar irradiation and ambient temperature for the next day are shown in Fig.3^8,39^. The available PV output power is estimated using Eq. (8)^10^. Where:$\:\:{I}_{s}$ is Solar irradiance (W/m^2^), $\:{P}_{STC}$ is the power retrieved from the PV array at the standard testing conditions (W/m^2^). $\:{T}_{c}$ is the module temperature (°C), $\:{\rho\:}_{PV}$ is the temperature coefficient of the module power (%/°C). The module temperature is given by Eq. (9)^10^, where:$\:\:{T}_{a}$ is the ambient temperature (°C) and $\:{T}_{n}$ is the nominal module temperature (°C).

8 $$\:{P}_{PV}={P}_{STC}\frac{{I}_{s}}{1000}\left(1+{\rho\:}_{PV}\left({T}_{c}-25\right)\right)$$

9 $$\:{T}_{c}={T}_{a}+\frac{{I}_{s}}{800}\left({T}_{n}-20\right)$$

The available PV power is shown in Fig. 2, which depicts the maximum available power from the PV system at any hour during the day. While the actual delivered power from the PV system is determined according to generation balance and operation constraints as shown later.

#### Power limits constraint

The battery drawn/delivered power shall be kept within limits to avoid excessive rate of charge/discharge. In addition, to avoid mechanical stress on the engine, the diesel generator load shall not be reduced below 20% of the rated value. On the other hand, the delivered power from the PV system is limited by the maximum available PV power. These conditions appear as inequality constraints as given by Eqs. (10−12)^33,40^.

10 $$\:{P}_{B\_min}\le\:{P}_{B}\left(t\right)\le\:{P}_{B\_max}$$

11 $$\:{P}_{dg\_min}\le\:{P}_{dg}\left(t\right)\le\:{P}_{dg\_max}$$

12 $$\:{P}_{PV\_min}\le\:{P}_{APV}\left(t\right)\le\:{P}_{PV\_max}$$

#### Battery energy storage constraint

To strength the battery life, the battery should be prevented from overcharging and deep discharging by adding constraints on the state of charge of the battery as given by Eq. (13)^8^. where: $\:{SOC}_{min}\:$ and $\:{SOC}_{max}$are the minimum and maximum state of charge of the battery.

13 $$\:{SOC}_{min}\le\:SOC\left(t\right)\le\:{SOC}_{max}\:$$

The SOC at the end of hour t is calculated from Eq. (14) or Eq. (15) according to the battery charging or discharging respectively^33,40^.

14 $$\:SOC\left(t+1\right)=SOC\left(t\right)+\:{\eta\:}_{ch}\frac{\:{P}_{B}\left(\text{t}\right){\Delta\:}\text{t}\:\:}{{W}_{n}}$$

15 $$\:SOC\left(t+1\right)=SOC\left(t\right)-\:\frac{\:{P}_{B}\left(t\right)\varDelta\:t\:\:}{{\eta\:}_{dis}{W}_{n}}$$

To maintain the battery available for next day operation, it is assumed the battery state of charge at the end of the day shall equal the battery state of charge at the beginning of the day as shown in Eq. (16)^33,40^.

16 $$\:{SOC}_{final}={SOC}_{initial}$$

#### Peak grid tie constraints

To ensure the system doesn’t violate the metering (available user electricity rate or the tie cable capacity), the maximum grid power shall be limited to the metering power as given by Eq. (17)^41^.

17 $$\:{P}_{g}\left(t\right)\le\:{P}_{meter}$$

### Demand side management constraints

In the optimal DSM, the system load is adapted to optimize the objective function. Therefore, there are additional constraints to the conventional EMS such as the permissible load variation, and the total energy per day.

#### Minimum and maximum load constraints

To enhance the flexibility of the proposed framework, the user has the option to adjust the upper and lower boundaries of the load during each interval as given by Eq. (18)^8^. Where $\:{P}_{L\_min}\left(t\right)$ and $\:{P}_{L\_max}\left(t\right)$ are the user’s minimum and maximum power at hour (t).

18 $$\:{P}_{L\_min}\left(t\right)\le\:{P}_{L}\left(t\right)\le\:{P}_{L\_max}\left(t\right)\:$$

The minimum load is determined from Eq. (19), Where $\:\mu\:\left(t\right)$ and $\:{P}_{Lo}\left(t\right)$ are the percentage of the flexible load and the original load at hour (t). The default value of $\:\mu\:\left(t\right)$ is assumed 0.25, which means only 25% pf the load can be shifted.

19 $$\:{P}_{L\_min}\left(t\right)=\left(1-\mu\:\left(t\right)\right){P}_{Lo}\left(t\right)$$

To avoid excessive loading during any interval due to load shifting, the permissible maximum power during each period is bounded by maximum load during the day before implementing demand response as given by Eq. (20). Where $\:{P}_{Lo}$ is vector of loading along the day as shown in Eq. (21).

20 $$\:{P}_{L\_max}\left(t\right)\le\:\text{m}\text{a}\text{x}\left({P}_{Lo}\right)$$

21 $$\:{P}_{Lo}={\left[\begin{array}{cccc}{P}_{Lo}\left(1\right)&\:{P}_{Lo}\left(1\right)&\:\dots\:&\:{P}_{Lo}\left(24\right)\end{array}\right]}^{T}$$

#### Daily energy constraints

The amount of load shifted from one interval shall be added to other intervals along the day. Hence, the total energy per day shall be the same as given (22).

22 $$\:\sum\:_{t=1}^{24}{P}_{L}\left(t\right)=\sum\:_{t=1}^{24}{P}_{Lo}\left(t\right)$$

### Consumer’s preferences

To enhance the proposed framework applicability, consumer’s preference concept is introduced to describe the user preferred operation strategy. Four consumer’s preferences operation modes are proposed, and user can select among them with various degree of flexibility.

#### Energy management

User selects this operation mode when he is sensitive for load rescheduling. During EM operation mode, the load is kept fixed which means DSM is not involved in the optimization process. This operation can be described as given by Eqs. (23) and (24). In EM mode, the proposed NG’s management system dispatches the generated power of the utility grid, the diesel generator, the battery, and the PV system to supply the load at minimum generation cost.

23 $$\:\mu\:\left(t\right)=0\:\forall\:\:t\in\:\left\{\text{1,2},\dots\:,\:24\right\}$$

24 $$\:{P}_{L}\left(t\right)={P}_{Lo}\left(t\right)\:\forall\:\:t\in\:\left\{\text{1,2},\dots\:,\:24\right\}$$

#### Energy management incorporating DSM with flexible intervals

During this mode, all intervals are assumed to have 25% flexible loads which can be shifted from one interval to another. In addition, all intervals are assumed flexible which means they can accommodate additional loads from the other intervals. Hence, Eqs. (25) and (26) show the constraints on loading intervals. Where $\:{P}_{Lo}$ is the vector consists of the load. In this mode, the proposed NG’s management system dispatches the power among different generation resources and reschedule the load to optimize the objective function.

25 $$\:\mu\:\left(t\right)=0.25\:\forall\:\:t\in\:\left\{\text{1,2},\dots\:,\:24\right\}$$

26 $$\:{P}_{L\_max}\left(t\right)\le\:\text{max}\left({P}_{Lo}\right)$$

#### Energy management incorporating DSM with non-flexible intervals

This mode provides the option for users to avoid load variation at predefined intervals which are called non-flexible intervals. Hence, the load during non-flexible intervals is kept at its original value as shown in Eq. (27), where $\:S$ is the set of non-flexible intervals. This restriction reduces the flexibility of the load which may increase the generation cost compared to full interval flexible interval. Without loss of generality, the non-flexible intervals are assumed at 7:00, 12:00 PM and at 18:00.

27 $$\:{P}_{L}\left(t\right)={P}_{Lo}\left(t\right)\:\forall\:\:t\in\:S$$

#### Energy management incorporating DSM and semi-flexible intervals

In this operation mode, the user defines the interval in which the load cannot be shifted while these intervals can accommodate additional loads from the other intervals. Hence, the minimum load during this interval is set by the original load as given by Eq. (28). Where $\:z$ is the set of semi-flexible intervals. Without loss of generality, the semi-flexible intervals are assumed at time 7:00, 12:00, and 18:00.

28 $$\:{P}_{L\_min}\left(t\right)={P}_{Lo}\left(t\right)\:\forall\:\:t\in\:z$$

## Hybrid MFO and Lagrange

**Fig. 4:**
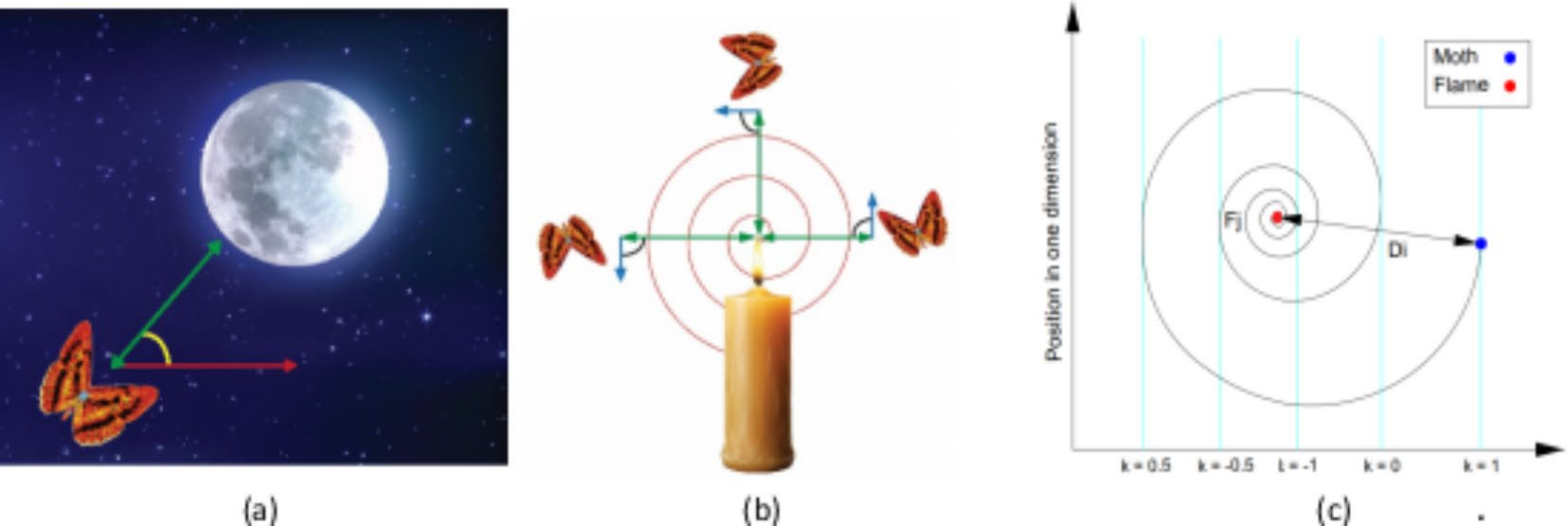
Moth-flame flight (a) fixed angle with the moon (b) artificial light (flame), (c) Logarithmic spiral of moth space around flame.

The MFO technique is natural inspired algorithm proposed by Seyedali Mirjalili^42^ to replicate the navigation of moths during night, which proved the superiority of MFO for optimizing several benchmark functions compared to several metaheuristic techniques^42^. MFO algorithm has been applied for several practical problems such as optimal dispatch and planning of microgrids in ref^43,44^, load management in ref^45^, and parameter estimation to escape of local minimum^46,47^. Moths fly in night by maintaining a fixed angle with respect to the moon for travelling in a straight line for long distances as shown in Fig. 4a. However, moths are unable to distinguish between the moon light and artificial lights therefore they are tricked by artificial lights such as flame. When moths see artificial light, they try to maintain a similar angle with the light to fly in straight line so, they are trapped in a useless/deadly spiral path around artificial lights as shown in Fig. 4b. in MFO, the candidate solutions are assumed the moths; the flying direction is the number of variables. The set of proposed moths are given in matrix M as shown in Eq. (29)^42^, where $\:n,d$ are the number of moths and the problem variables (dimensions). OM is the fitness vector corresponding to the Moths matrix M.

29 $$M = \left[ {\begin{array}{*{20}c} {m_{{1,1}} } & {m_{{1,2}} } & \cdots & \cdots & {m_{{1,d}} } \\ {m_{{2,1}} } & {m_{{2,2}} } & \cdots & \cdots & {m_{{2,d}} } \\ \vdots & \vdots & \vdots & \vdots & \vdots \\ \vdots & \vdots & \vdots & \vdots & \vdots \\ {m_{{n,1}} } & {m_{{n,2}} } & \cdots & \cdots & {m_{{n,d}} } \\ \end{array} } \right],~~OM = \left[ {\begin{array}{*{20}c} {OM_{1} } \\ {OM_{2} } \\ \vdots \\ \vdots \\ {OM_{n} } \\ \end{array} } \right]$$

For each moth there is corresponding flame, therefore there are$\:\:N$ flames. Each flame is located in the space coordinated by the problem directions; thus, the flame matrix has the same dimensions as moth matrix as given by Eq. (30)^42^. Where: $\:OF$ is the fitness vector corresponding to the flame matrix $\:F$

30 $$F = \left[ {\begin{array}{*{20}c} {f_{{1,1}} } & {f_{{1,2}} } & \cdots & \cdots & {f_{{1,d}} } \\ {f_{{2,1}} } & {f_{{2,2}} } & \cdots & \cdots & {f_{{2,d}} } \\ \vdots & \vdots & \vdots & \vdots & \vdots \\ \vdots & \vdots & \vdots & \vdots & \vdots \\ {f_{{n,1}} } & {f_{{n,2}} } & \cdots & \cdots & {f_{{n,d}} } \\ \end{array} } \right],~~OF = \left[ {\begin{array}{*{20}c} {OF_{1} } \\ {OF_{2} } \\ \vdots \\ \vdots \\ {OF_{n} } \\ \end{array} } \right]$$

Moths and flames are both solutions to the problem, while Moths are search agents to determine the best solutions, and flames represent the best solutions obtained by moths. These best solutions represent flags for moths, therefore each Moth search around the flames and update its position to determine better solution. The moth travels in a spiral shape around the flame as shown in Fig. 4c, which is described by logarithm function that start at the moth position and end at the flame. To avoid trapping in the local minimum, each moth updates its position considering only one of the flames as shown in Eq. (31)^43,44^. Where; $\:{D}_{i}$ is the distance from moth $\:i\:$and flame$\:\:j$, $\:b$ is a constant that controls the shape of spiral, and $\:k\in\:[-\text{1,1}]$ is a random number. The distance $\:{D}_{i}$ is calculated using Eq. (32)^43,44^. Eventually, the flame is updated if the moth is better fitted than its flame.

31 $$\:{M}_{i}={D}_{i}{e}^{bk}\text{cos}\left(2\pi\:k\right)+{F}_{j}$$

32 $$\:{D}_{i}=\left|{F}_{j}-{M}_{i}\right|$$

The presence of large number of flames may deteriorate the exploitation process of MFO, therefore the number of flames is decreased along the search space as given by Eq. (33)^43^. Where: $\:T$ is the total number of iterations, $\:l$ is the current iteration number, and $\:n$ is the initial number of flames (or number of Moths). Hencs, at the begging, the number of flames is $\:N$, while at end of iterations there is only one flame corresponding to the best solution.

33 $$\:{N}_{F}=round\left(n-l\frac{n-1}{T}\right)$$

**Fig. 5:**
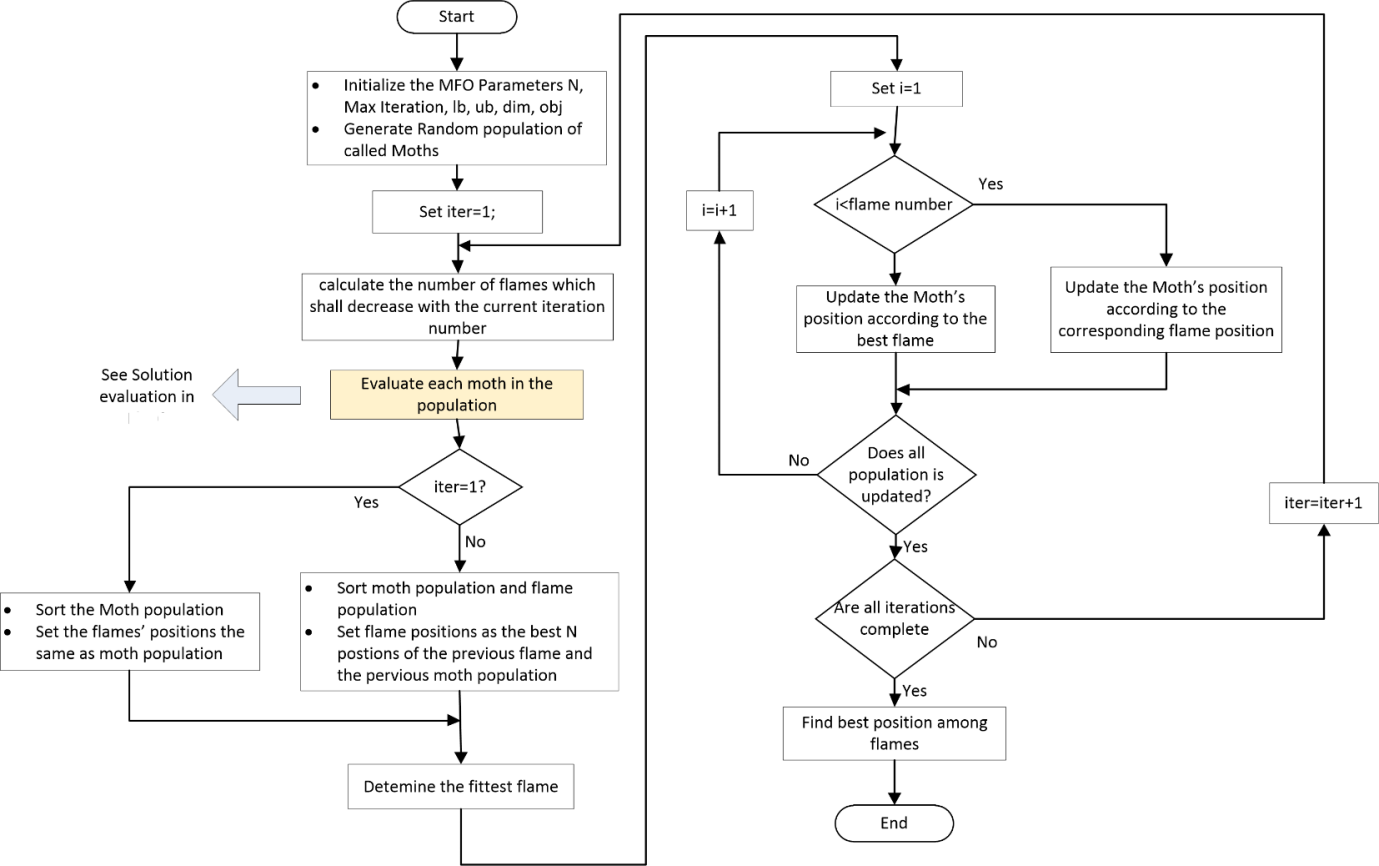
Flowchart of the Moth-flame optimization technique.

The moths search space is bounded by lower ($\:{l}_{b}$) and upper ($\:{u}_{b}$) boundaries, where $\:{u}_{i}$ and $\:{l}_{i}$ are the upper and lower boundaries of moth $\:i$ as shown in Eqs. (34) and (35)^42^, respectively. Figure 5 shows the flowchart of the MFO.

34 $$\:{u}_{b}=\left\{{u}_{1},{u}_{2},\dots\:,{u}_{d-1},\:{u}_{d}\right\}$$

35 $$\:{l}_{b}=\left\{{l}_{1},{l}_{2},\dots\:,{l}_{d-1},\:{l}_{d}\right\}$$

**Fig. 6:**
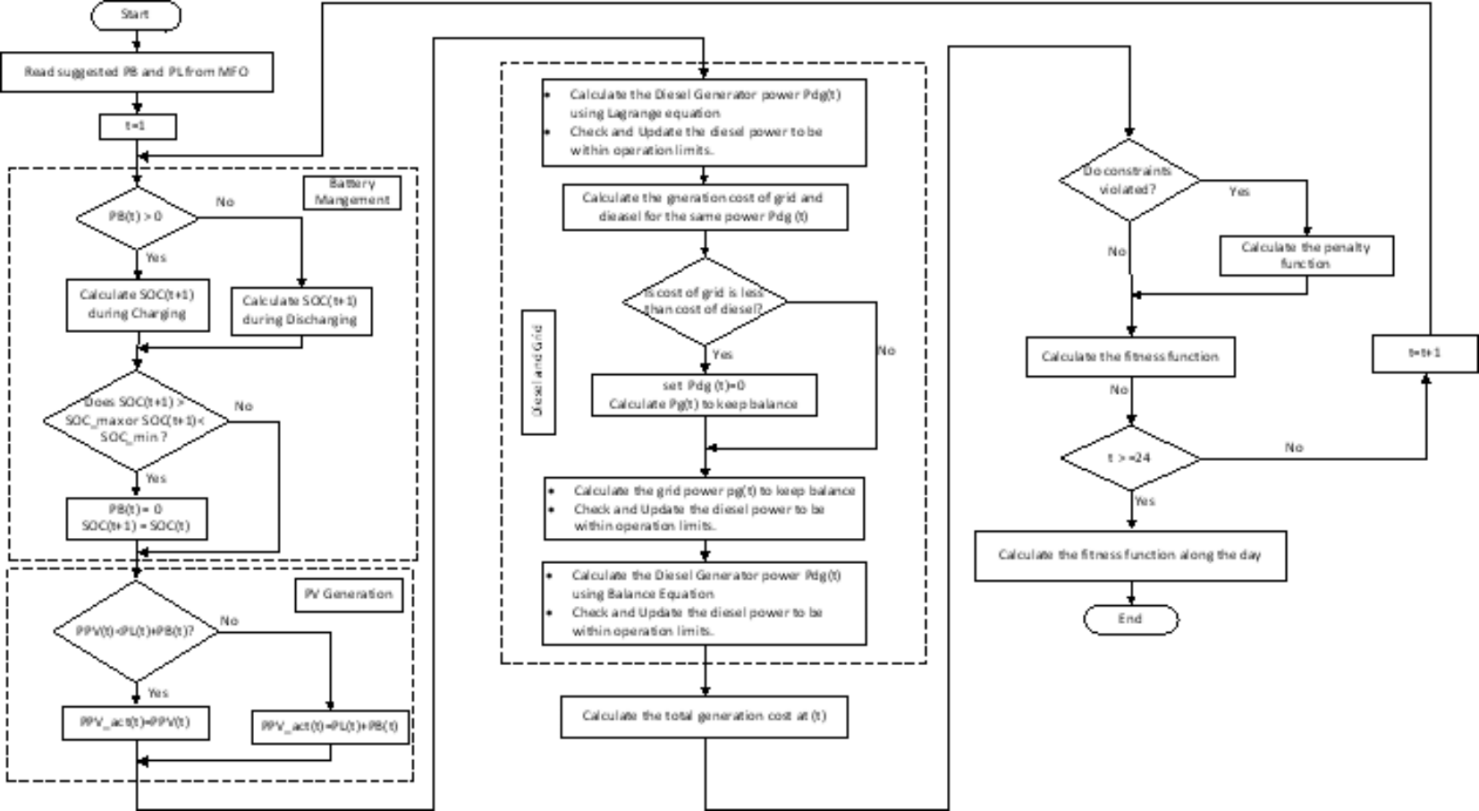
Evaluation of each solution (Moth).

 The evaluation of each proposed solution by the MFO is determined using the flow chart of Fig. 6. The optimization variables in the proposed NG are the power of Diesel, battery, grid, PV, and load. Each decision variable is a vector of 24 values along the day. Hence the number of variables becomes huge. In addition, metaheuristic technique may strap in local minimum. Hence, to increase the possibility for global optimum solution, some treatment is employed to minimize the number of decision variables which shall be determined by the MFO (battery and load power only).

1. As shown in Fig. 6 of PV generation subroutine, if the PV power is less than the load and battery hence all the PV generated power is utilized. On the other hand, if the PV generated power is greater than the load and battery, the PV power shall be curtailed to the sum of load and battery consumption.
2. When the grid and diesel are working together, Lagrange multiplier can be involved to optimize the operation, in which the incremental generation cost of the grid and diesel shall be equal as given by Eq. (36)^8^. From the grid and diesel generation cost and Eq. (36), the required power of the DG is given by Eq. (37). Furthermore, the final decision of the DG shall satisfy the boundaries constraints.

36 $$\:\frac{\partial\:{C}_{dg}\left(t\right)}{\partial\:{P}_{dg}\left(t\right)}=\frac{\partial\:{C}_{g}\left(t\right)}{\partial\:{P}_{g}\left(t\right)}$$

37 $$\:{P}_{dg}\left(t\right)\:=\frac{{\rho\:}_{g}\left(t\right)-b}{2a}$$

3. The grid power is determined to keep the balance between generation and demand

Note that, Lagrange multiplier method provides the optimal solution when both the diesel and grid are working. While the system may optimal if the diesel or grid off. To handle this discontinuity of the variable, additional checks are added to decide what is optimal Lagrange or getting diesel or grid off as shown in diesel and grid subroutine of Fig. 6.

## Study cases

**Fig. 7:**
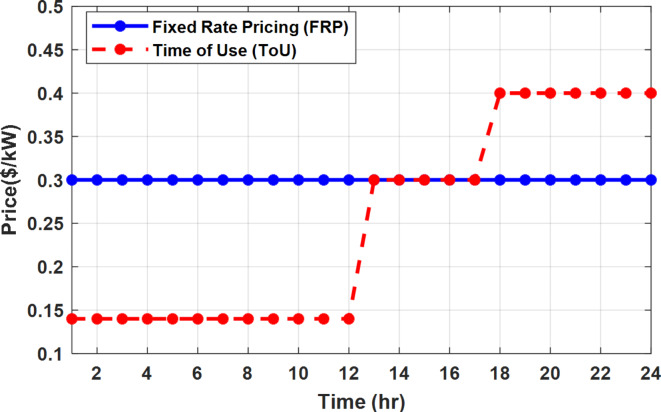
Different grid strategies (FRP and ToU).

**Fig. 8:**
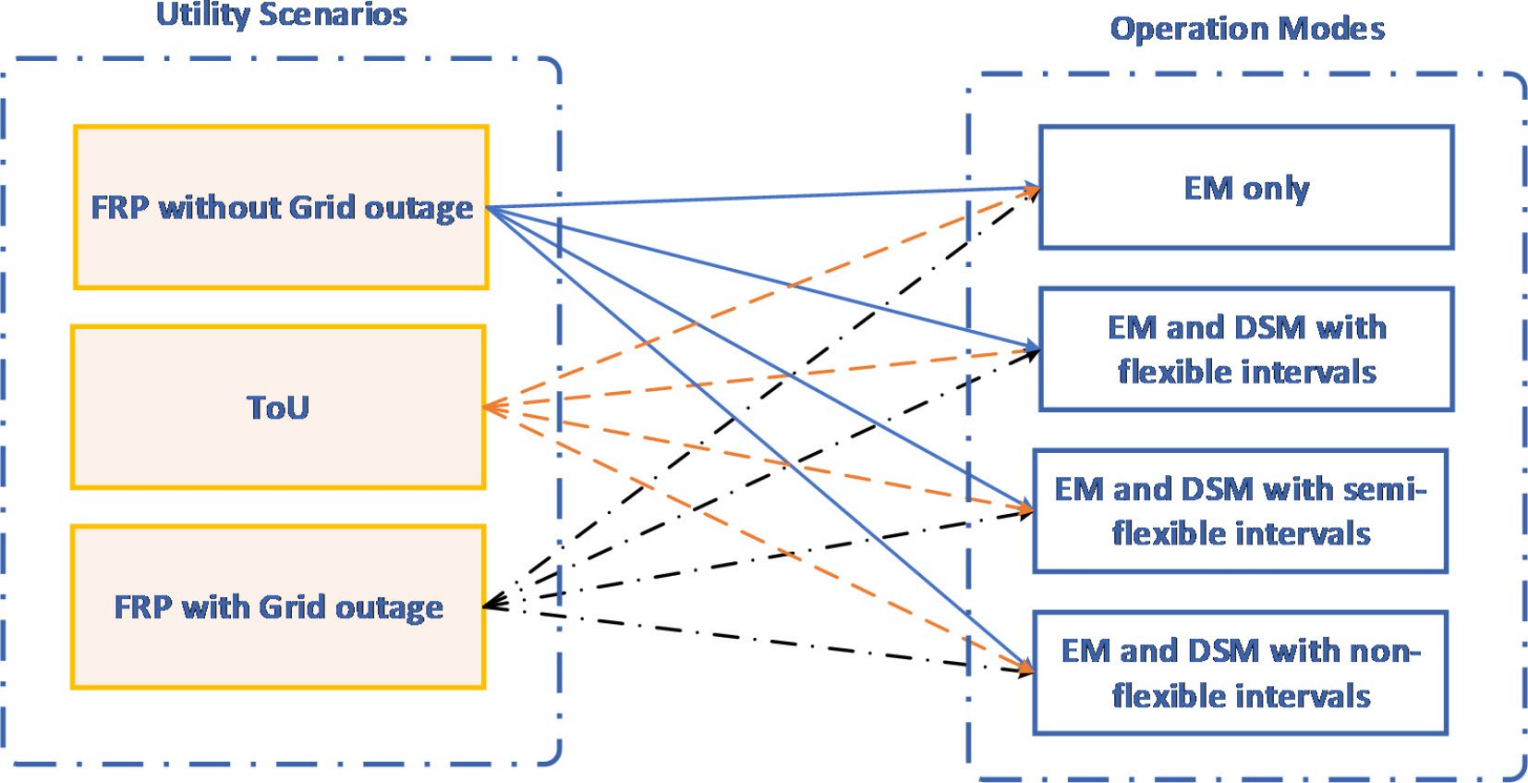
The configuration of the study cases.

The system performance is investigated under different grid strategies as shown in Fig. 7 such as fixed rate pricing (FRP), time of use pricing (ToU), and flat rate pricing (FRP) with grid outage. Each grid strategy is combined with one of the users selected operation mode known as EM, EM and DSM with flexible intervals, EM and DSM with semi-flexible interval, and EM and with non-flexible intervals as shown in Fig. 8.

 In each grid strategy, the user preferences are compared with respect to:

1. The total generation cost involves the cost of grid power, diesel generation and battery as given by Eq. (1).
2. The percentage of energy not supplied ($\:{E}_{N.S}$) as given in Eq. (38), which represents the amount of load curtailment due to shortage of generation such as grid outage scenario.
3. The amount of renewable energy curtailed (REC) as given by Eq. (39), which illustrates the utilization of the installed PV system.

38 $$\:{E}_{N.S}\left(\%\right)=100\left(1-\sum\:_{t=1}^{24}{P}_{L\_act}\left(t\right)/\sum\:_{t=1}^{24}{P}_{L\_o}\left(t\right)\right)$$

39 $$\:REC\left(\%\right)=100\left(1-\sum\:_{t=1}^{24}{P}_{PV\_ouput}\left(t\right)/\sum\:_{t=1}^{24}{P}_{PV}\left(t\right)\right)$$

### Flat rate pricing strategy

**Fig. 9:**
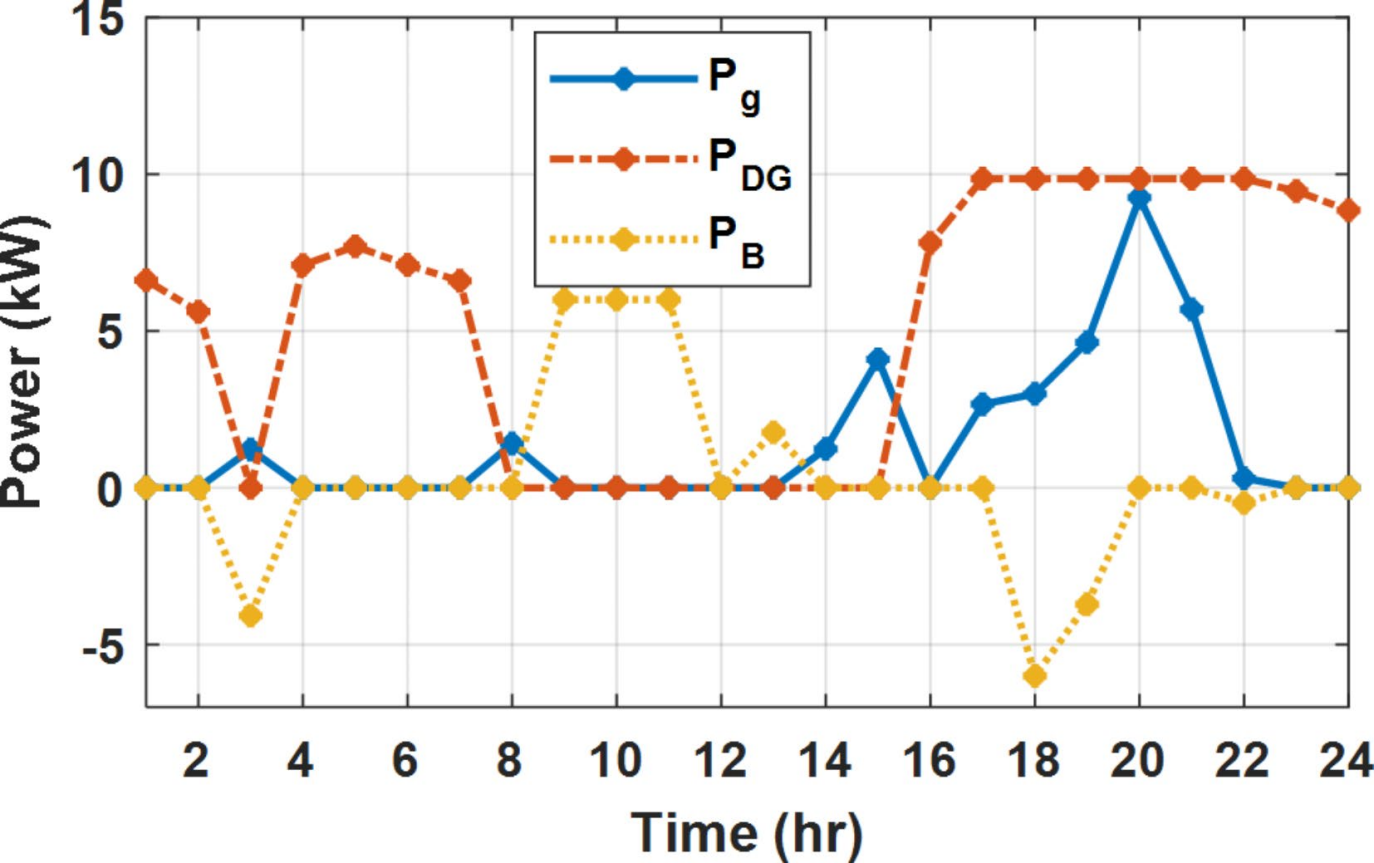
Utility grid, DG, and battery power flow during FRP without DSM.

**Fig. 10:**
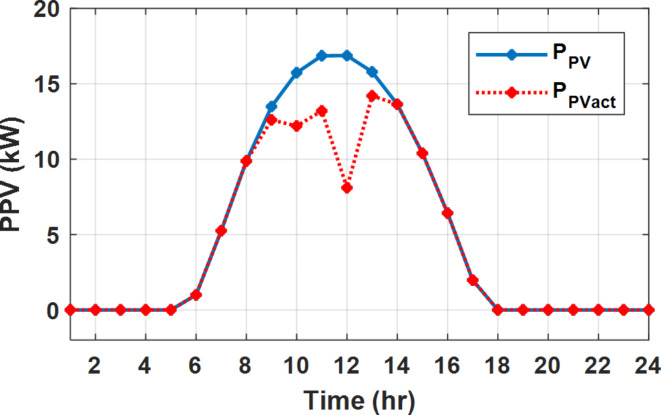
Expected and actual PV power flow during FRP without DSM.

**Fig. 11:**
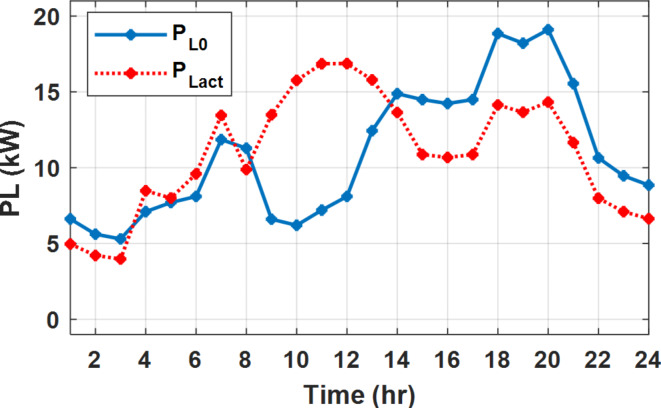
Load demand during FRP with/out DSM and flexible intervals.

**Fig. 12:**
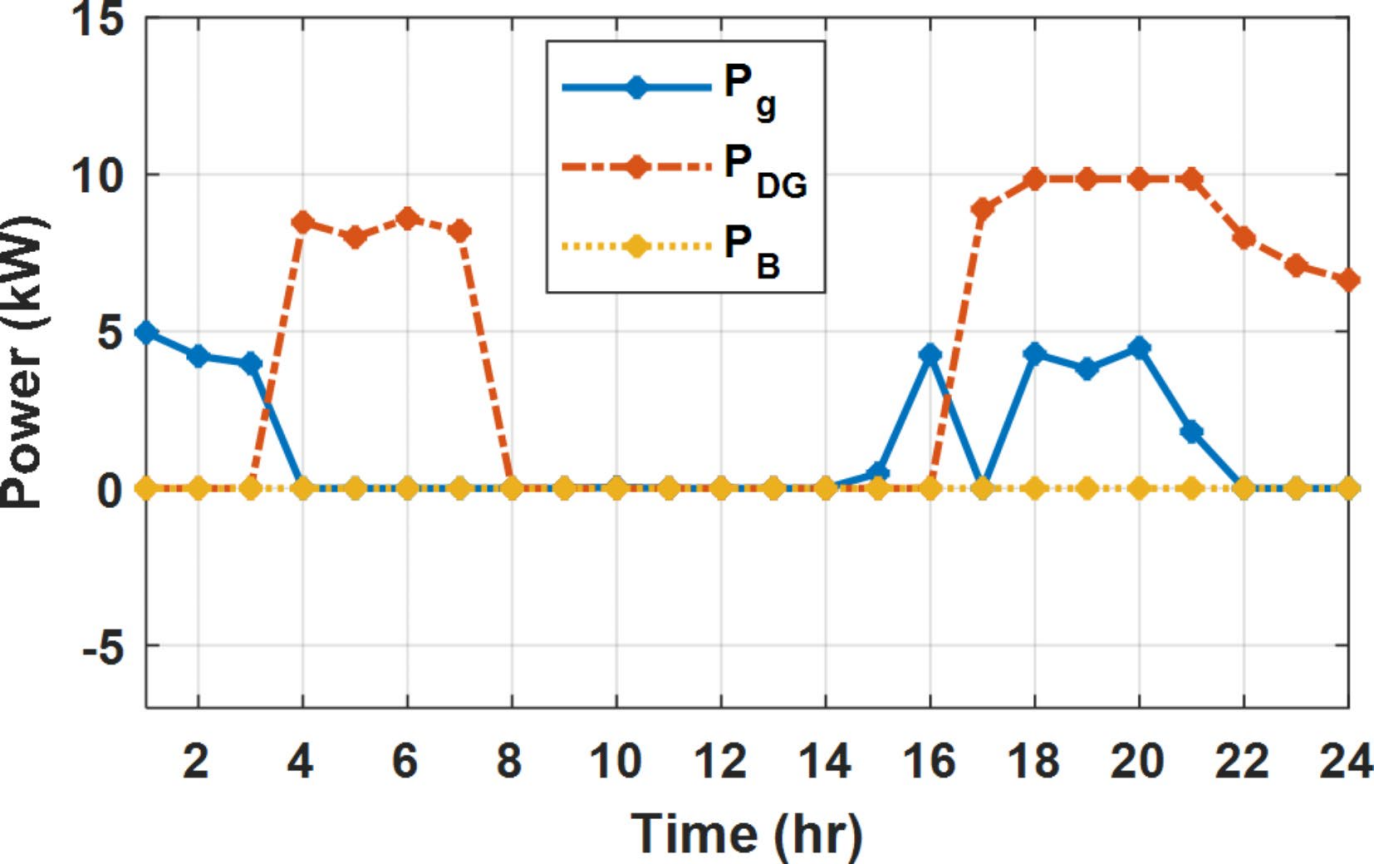
Utility grid, DG, and battery power flow during FRP with DSM full flexible.

**Fig. 13:**
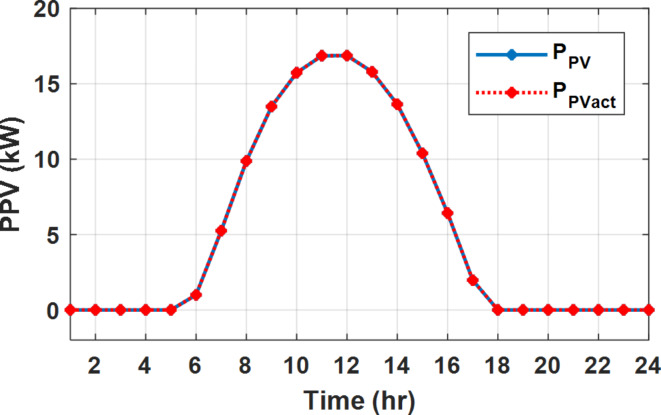
Expected and actual PV power flow during FRP with DSM full flexible.

**Fig. 14:**
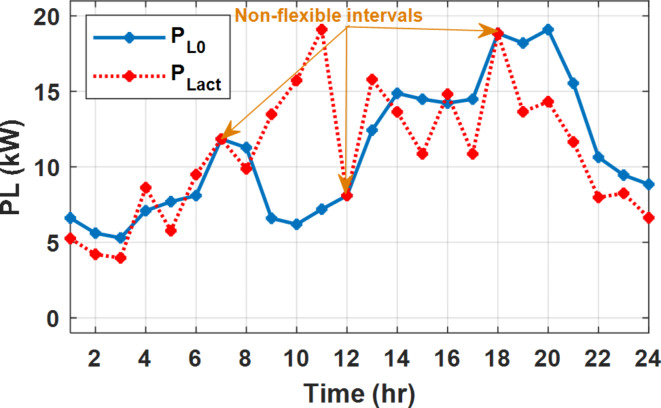
Load demand during FRP with/out DSM and non-flexible intervals.

**Fig. 15:**
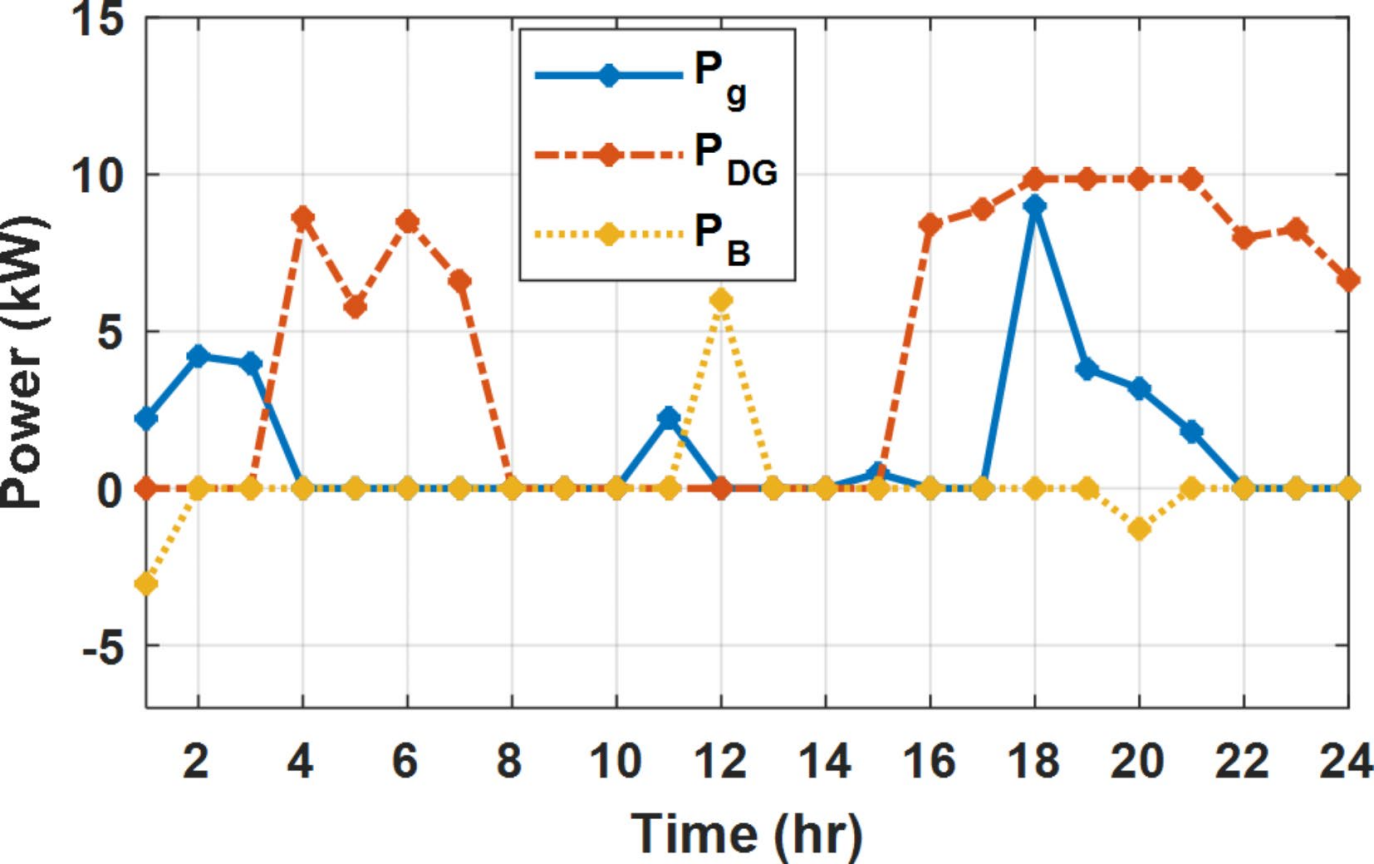
Utility grid, DG, and Battery Power flow during FRP with DSM and non-flexible intervals.

**Fig. 16:**
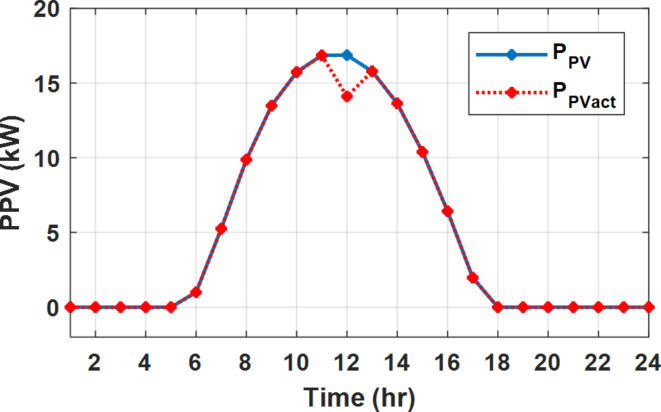
Expected and Actual PV Power flow during FRP with DSM and non-flexible intervals.

**Fig. 17:**
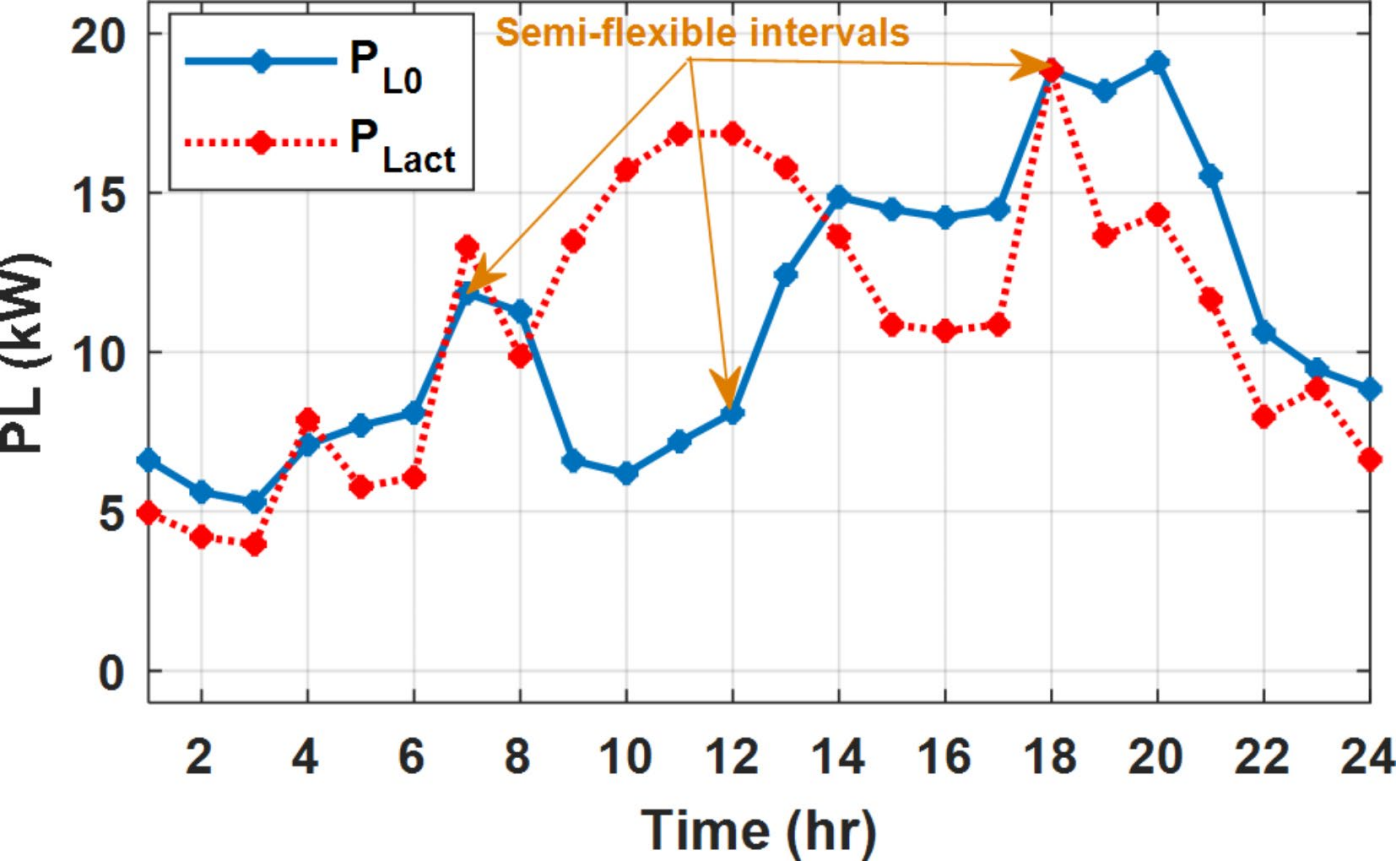
Load demand during FRP with/out DSM and semi-flexible intervals.

**Fig. 18:**
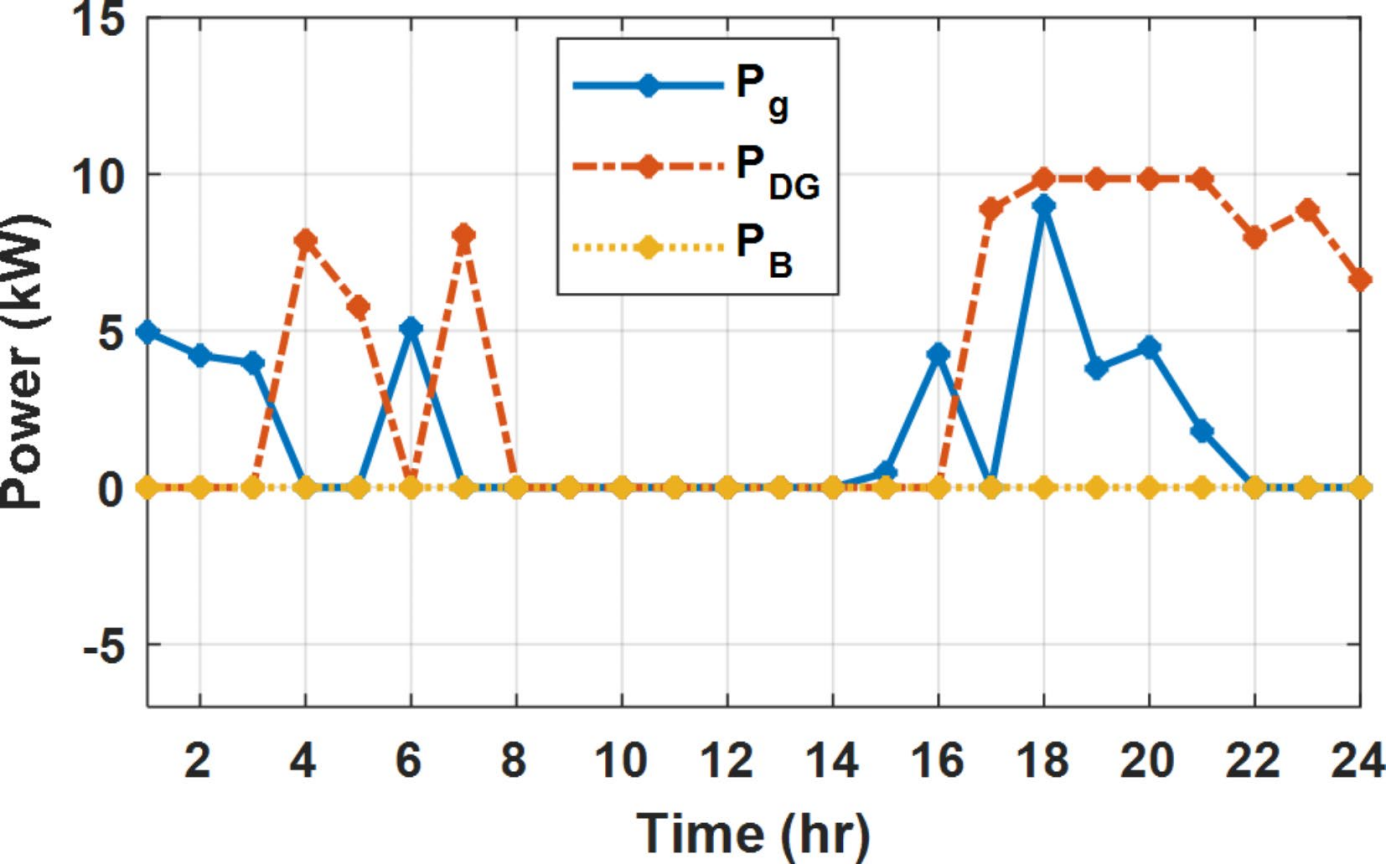
Utility grid, DG, and Battery Power flow during FRP with DSM and semi-flexible intervals.

**Fig. 19:**
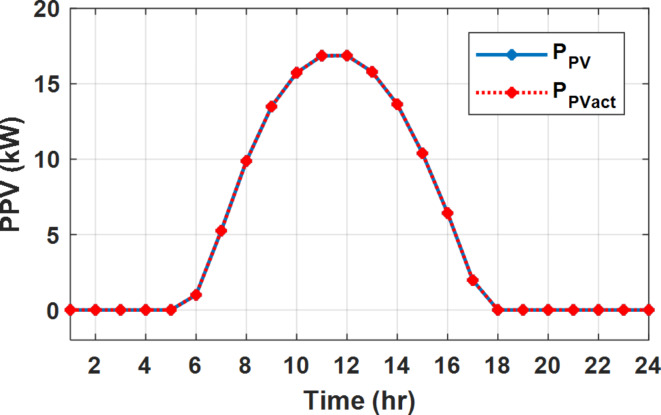
Expected and Actual PV Power flow during FRP with DSM and semi-flexible intervals.

During flat rate pricing, the utility electricity price is fixed at 0.3 $/kW throughout the day as shown in Fig. 7. With EMS only, the battery is utilized to store part of generation surplus from the PV, when the PV generation becomes greater than the NG demand as shown in Fig. 9. However, due to limited capacity of the battery and prevention of power injection into the grid, the system is unable to extract all available power from the PV as shown in Fig. 10. This causes PV energy curtailment of about 14.48% (Figs. 11, 12, 13, 14, 15, 16, 17, 18 and 19). On the other hand using EM and DSM with flexible intervals along the day, the system can shift the load from the rest periods of the day to the interval which has high availability of solar as shown in Fig. 11. So, the total energy produced from PV is efficiently utilized as shown in Fig. 13 which provides a significant reduction in the generation cost. With the presence of non-flexible intervals (7:00, 12:00, and 18:00), the modified load during these non-flexible intervals after implementing DSM is the same as the original load as shown in Fig. 14. This restriction reduces the extracted energy from the PV system as shown in Fig. 16 and reduces the enhancement of the generation cost. On the other hand, with semi-flexible intervals, the operation provides more flexibility in the new load compared to non-flexible load as shown in Fig. 17 which leads to extracting all the generated energy of the PV systems shown in Fig. 19. It is clear, with a fixed tariff. When DG and grid are operating in the same interval, the DG produces a fixed amount of power to maintain optimal operation according to Lagrange method as shown in Figs. 9, 12, 15 and 18.

**Fig. 20:**
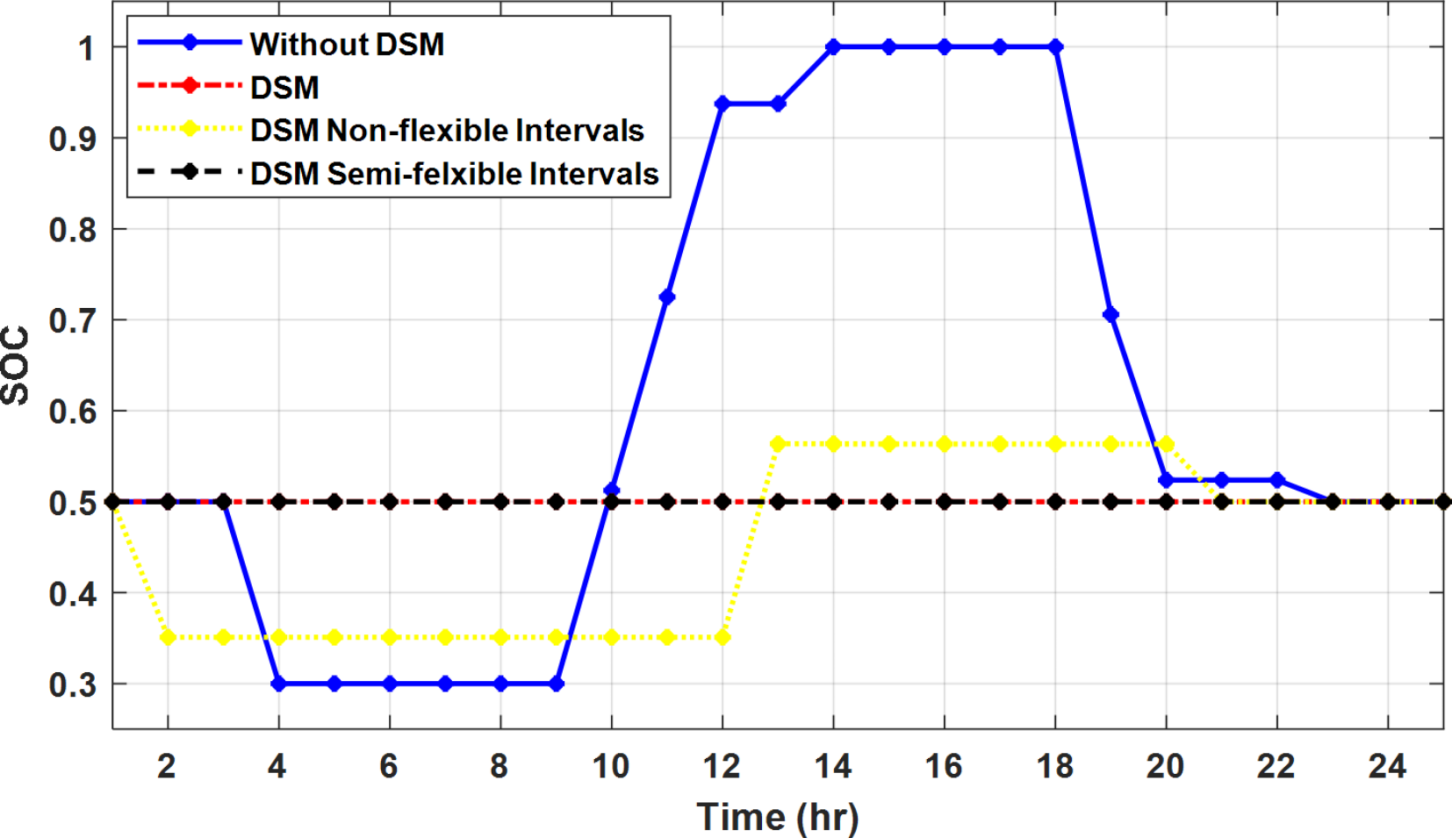
Battery SOC during different operation modes with fixed rate strategy.

The DSM reduces the required energy from the battery significantly is shown in Fig. 20, which reduces the required battery size. It is clear, with EM only, battery utilization is significantly effective to store the surplus of renewable generation in the interval of high PV generation and discharge in the interval of solar absence. While with full flexible and semi-flexible intervals, the system can work optimally without battery which leads to minimization of the system installation cost and required space. With non-flexible DSM, the battery charges at solar surplus intervals because when the non-flexible intervals occur during solar peak (12:00). Even though the system becomes non-flexible the required energy from the battery is less compared to operation without DSM.

The summary of fixed tariff operation is provided in Table 3. The total energy cost with EM only is about 48.63 $/day and renewable curtailment is about 14.478%. The EM with DSM, with flexible intervals, reduces the generation cost to 38.41 $/day (saving of 10.22 $) which leads to 21.02% cost reduction. With non-flexible intervals, the generation cost reaches 40.74 $/day (saving of 7.9 $/day) and cost reduction of 16.24% compared to operation without DSM with renewable curtailment is about 2.17%. Applying semi-flexible intervals scenario, the generation cost is reduced from 48.63 $/day to 38.71$/day which means 20.41% reduction in generation cost compared to without DSM with full utilization of the renewable generation. It shall be mentioned, renewable energy curtailment occurs due to limits of the storage battery in term of storage capacity and rate of energy charge, so this curtailment can be minimized while the system becomes larger and costly.

**Table 3:** Performance indicators during flat rate pricing strategy.

	EM only (without DSM)	EM and DSM (with flexible intervals)	EM and DSM (with non-flexible intervals) (7:00, 12:00, and 18:00)	EM and DSM (with semi-flexible intervals) (7:00, 12:00, and 18:00)
Total generation Cost ($)	48.63 $	38.41 $	40.74 $	38.71 $
Total Cost reduction	-	21.02% (10.22 $)	16.24% (7.9 $)	20.41% (9.92 $)
Energy not supplied (%)	0%	0%	0%	0%
Renewable Energy Curtailment (%)	14.48%	0%	2.17%	0%

### Time of use pricing strategy

The most common ToU strategy divides the operation along the day into three segments known as low, off-peak, and peak intervals^48^. The electricity price during each interval is assumed 0.14 $/kWh (01:00 to 13:00), 0.3 $/kWh (13:00 to 18:00), and 0.4 $/kWh (18:00 to 01:00) as shown in Fig. 7 for low, off-peak, and peak intervals respectively similar to ref^48^.

**Fig. 21:**
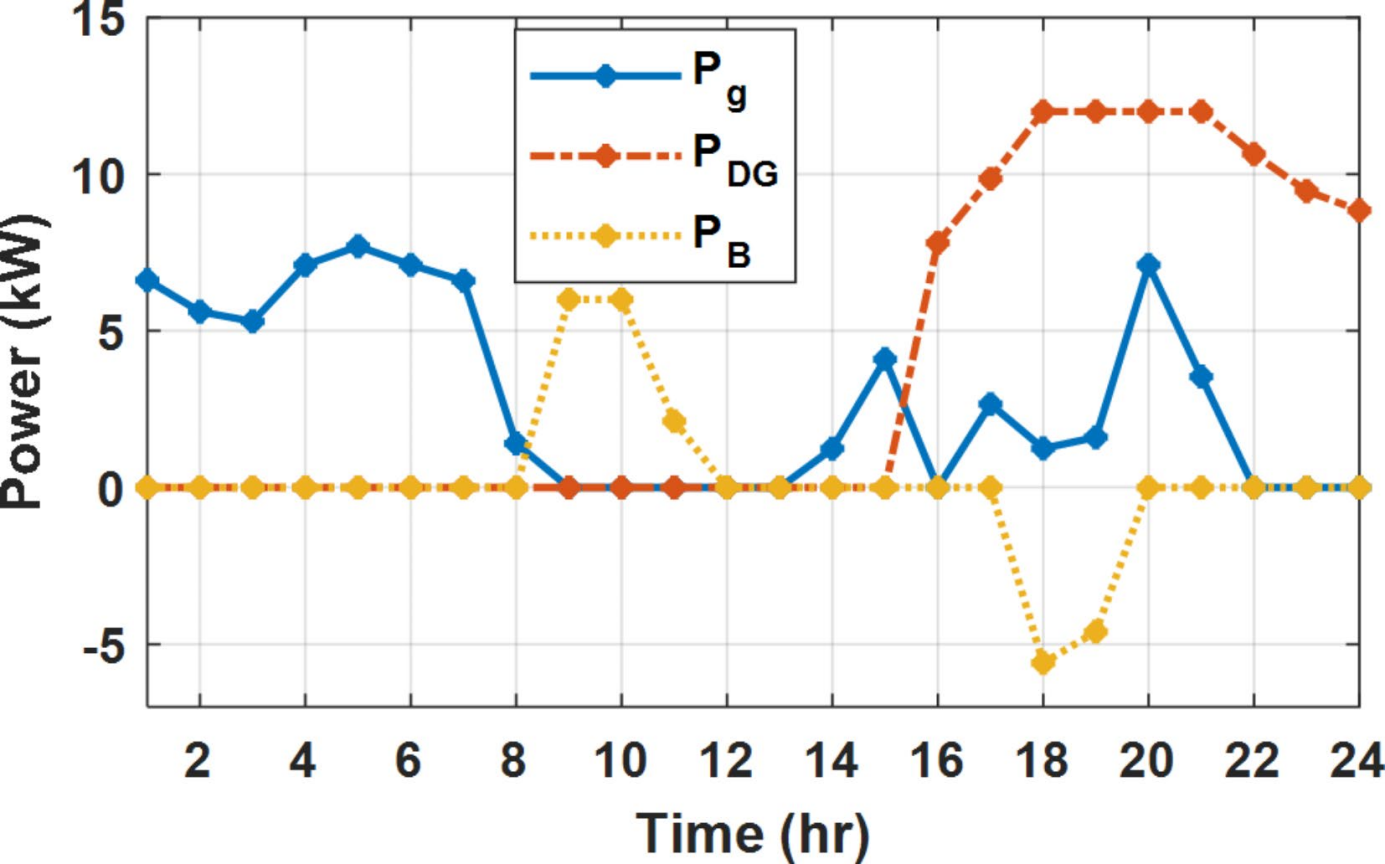
Utility grid, DG, and battery power flow during ToU without DSM.

**Fig. 22:**
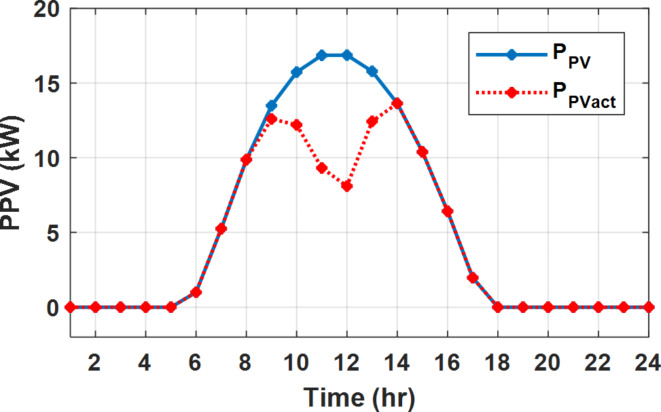
Expected and actual PV power flow during ToU without DSM.

**Fig. 23:**
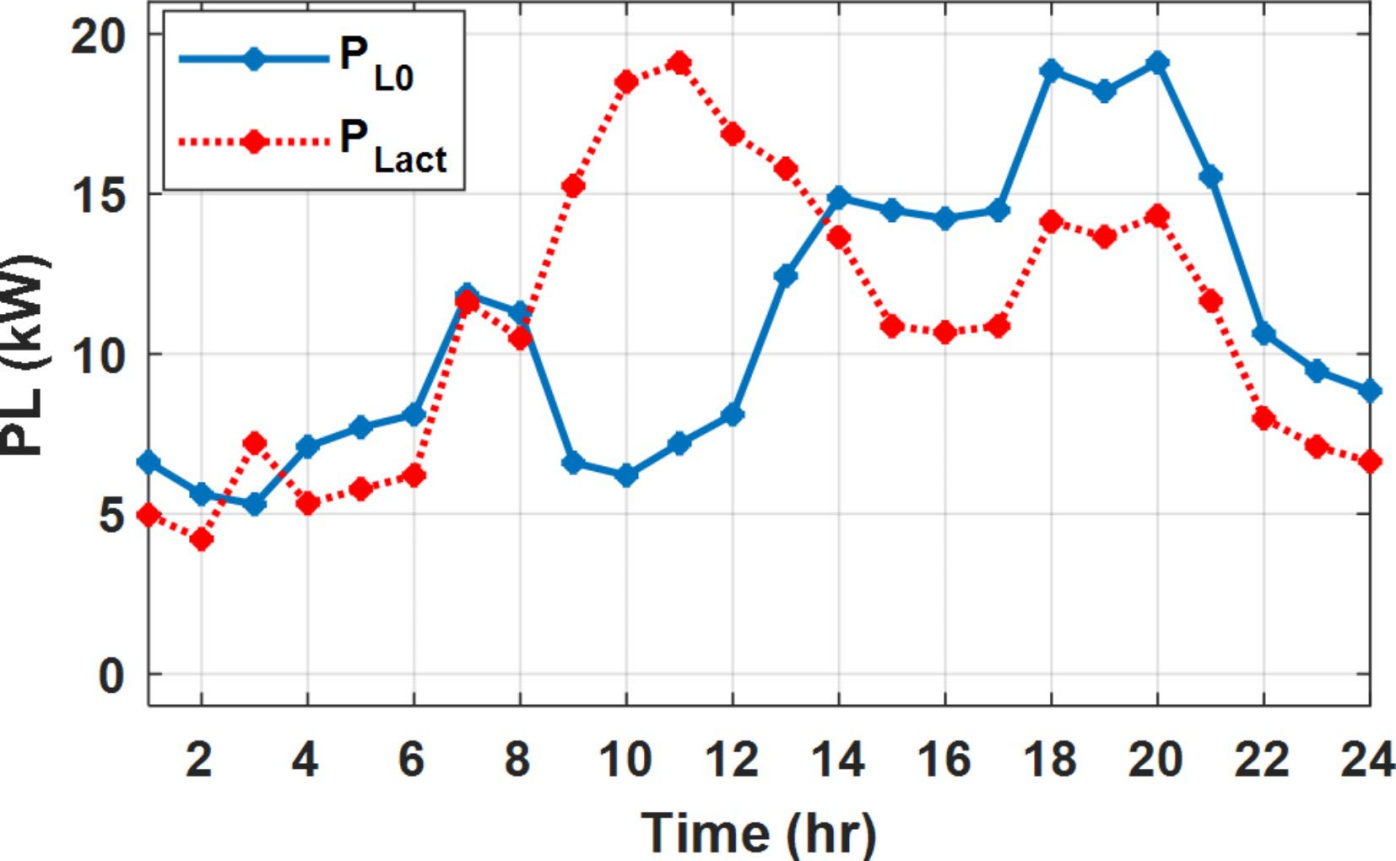
Load demand during ToU with/out DSM and flexible intervals.

**Fig. 24:**
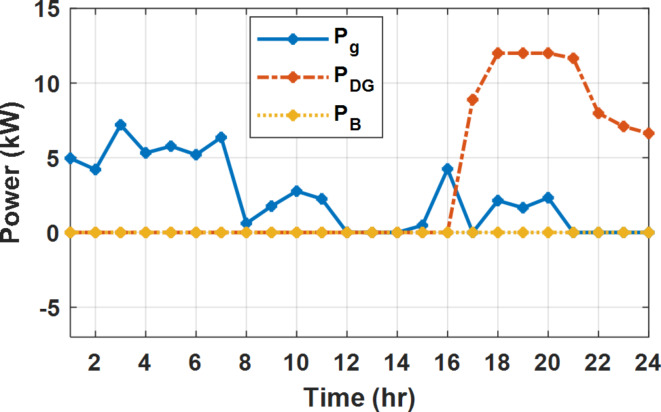
Utility grid, DG, and battery power flow during ToU with DSM full flexible.

**Fig. 25:**
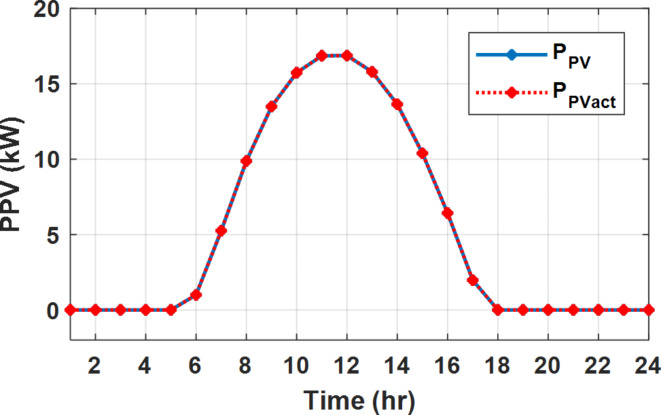
Expected and actual PV power flow during ToU with DSM full flexible.

**Fig. 26:**
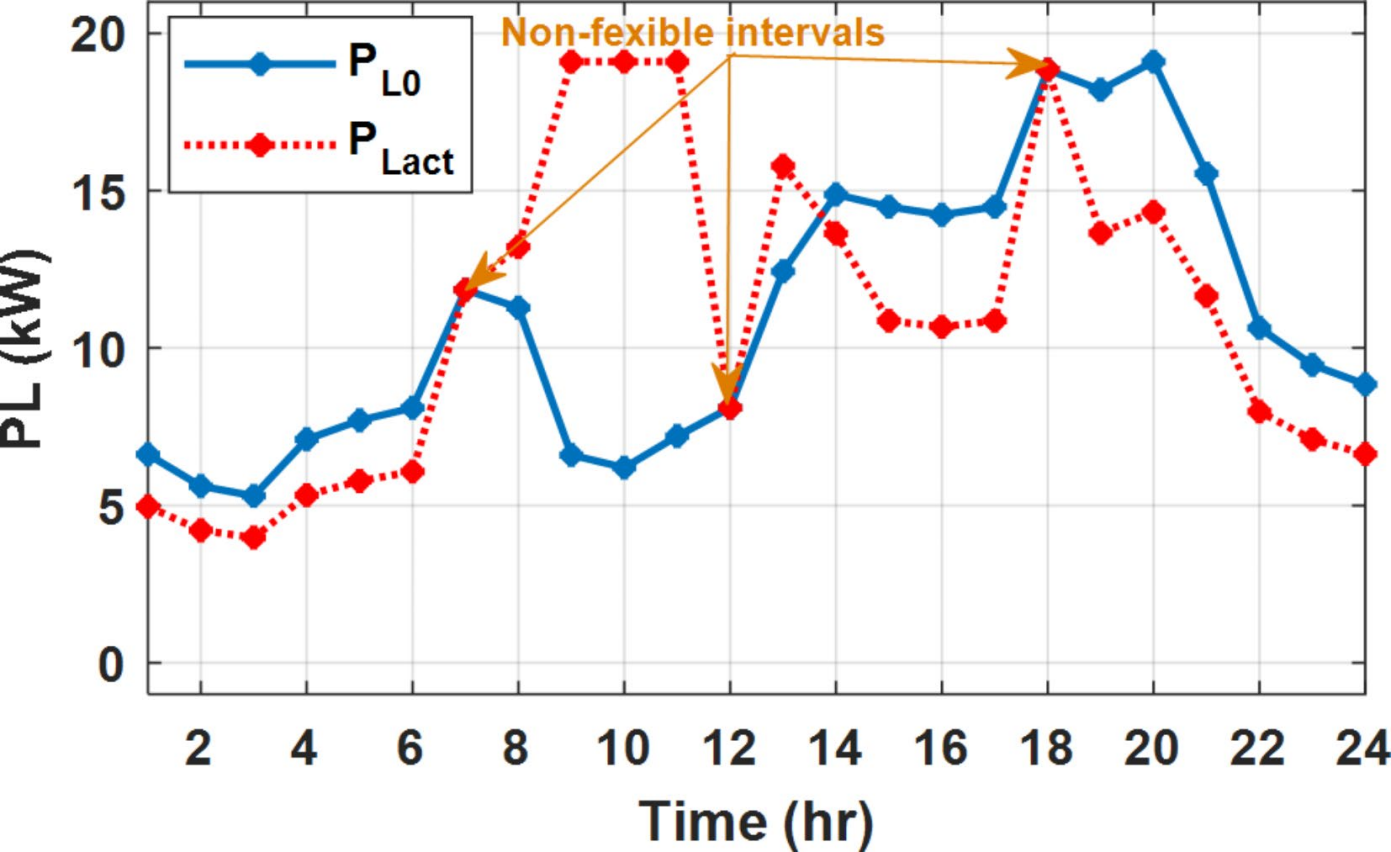
Load demand during ToU with/out DSM and non-flexible intervals.

**Fig. 27:**
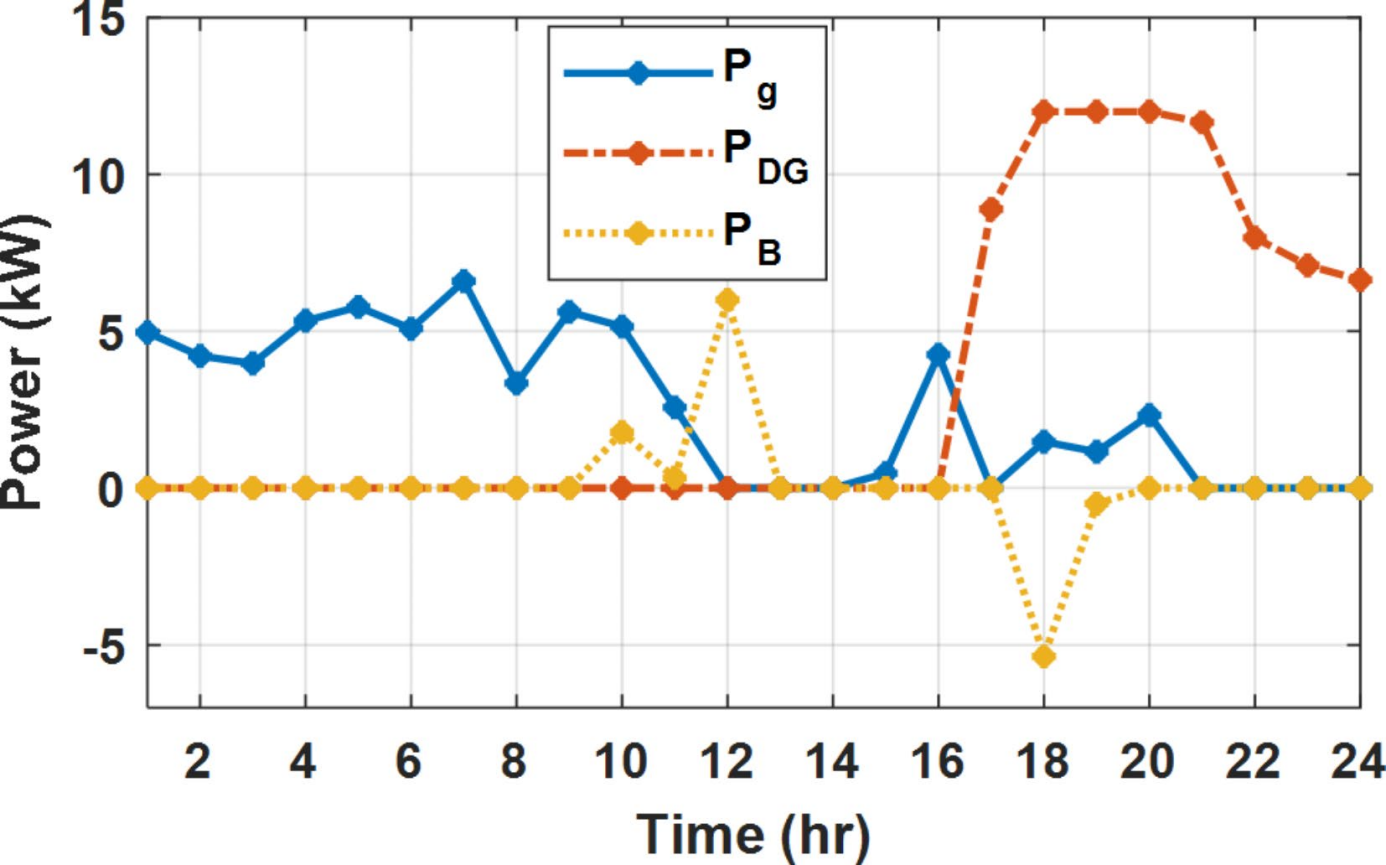
Utility grid, DG, and battery power flow during ToU with DSM and non-flexible intervals.

**Fig. 28:**
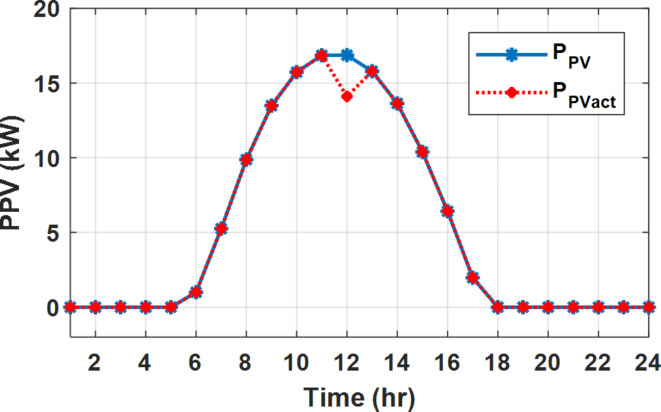
Expected and actual PV power flow during ToU with DSM and non-flexible intervals.

**Fig. 29:**
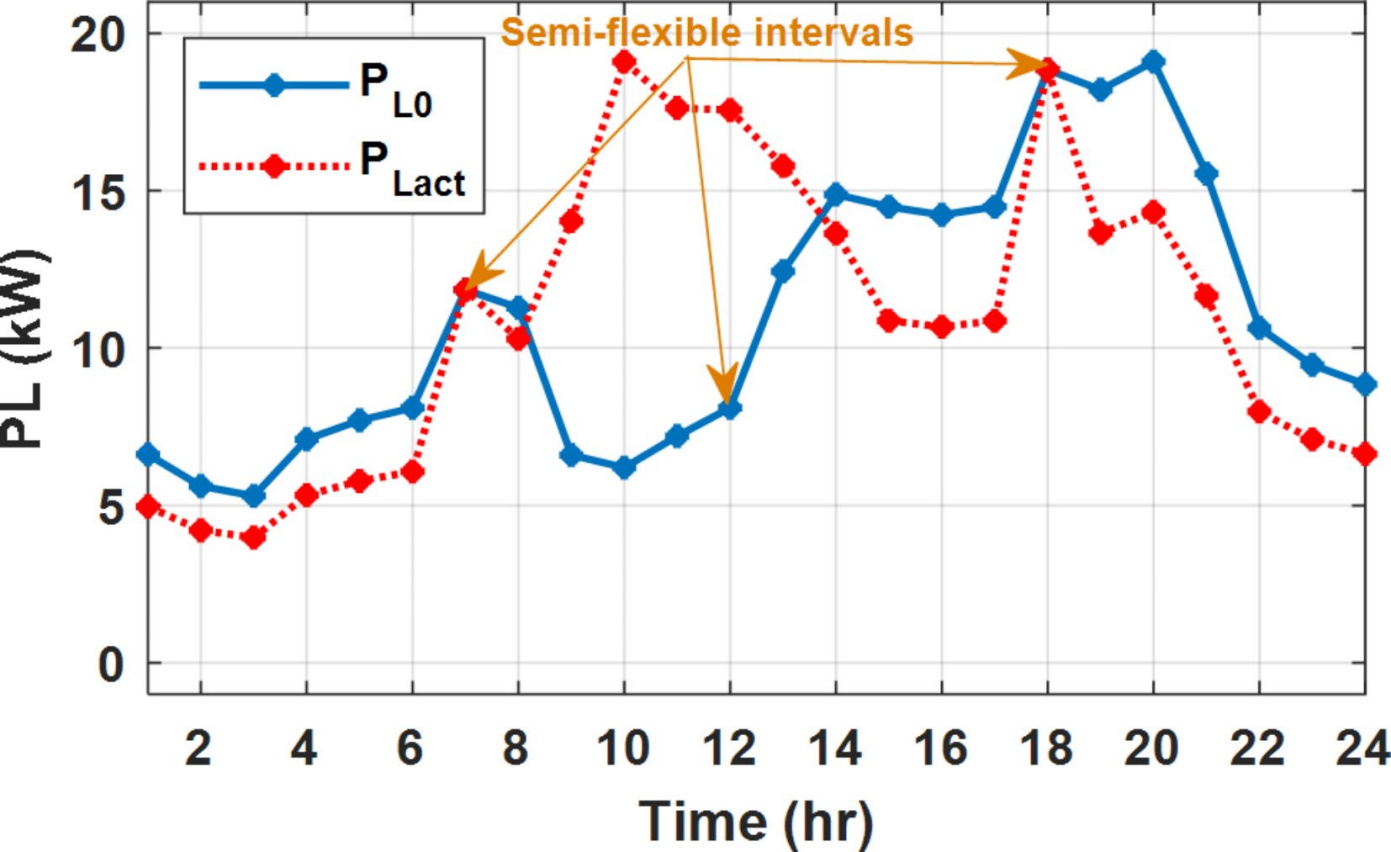
Load demand during ToU with/out DSM and semi-flexible intervals.

**Fig. 30:**
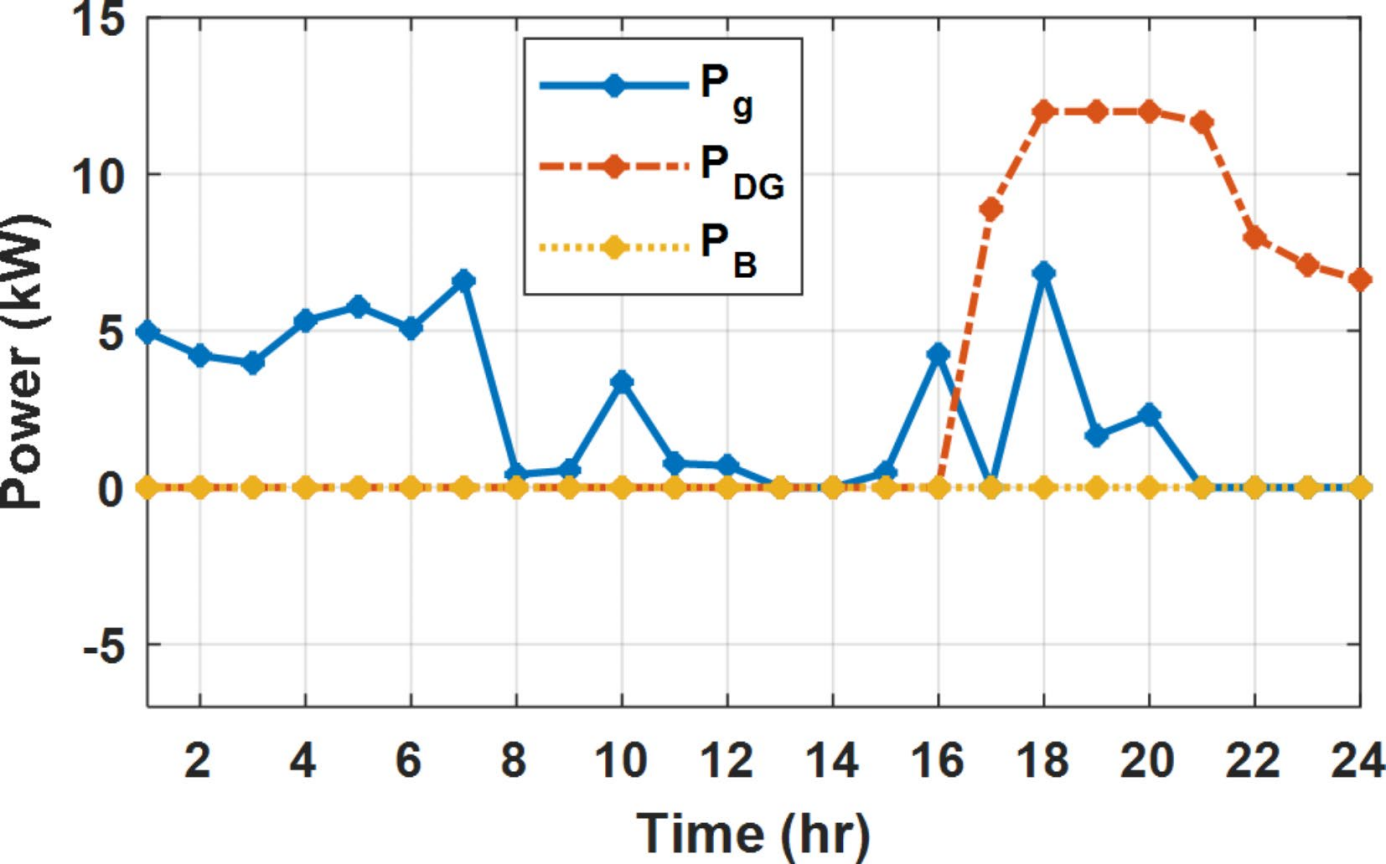
Utility grid, DG, and battery power flow during ToU with DSM and semi-flexible intervals.

**Fig. 31:**
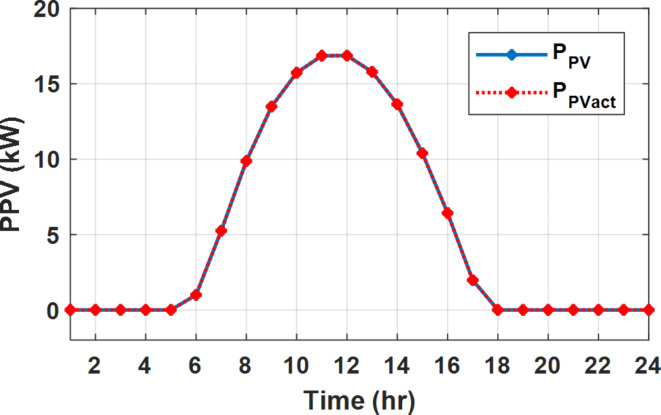
Expected and actual PV power flow during ToU with DSM and semi-flexible intervals.

With EM only, the battery charges during high solar availability and discharges in the night during high pricing period as shown in Fig. 21. During high tariff intervals at the end of the day, the grid energy supply is reduced and the diesel generator operates as shown in Fig. 21. Moreover, due to limited capacity of battery and lack of power injection into the grid, the PV generation is curtailed to keep balance as shown in Fig. 22, which raises the generation cost compared with DSM operation. Selecting EM and DSM with flexible intervals, the load is increased during peaks of solar irradiance because this interval is the least electricity price when considering the PV as a source of generation with low operation cost. While the load is decreased during high electricity price interval as shown in Fig. 23. Also, the diesel generator operates during high price period to minimize the request energy of the utility grid as shown in Fig. 24. It shall be noted, consuming the PV power into the load instead of charging/discharging the battery is more economic (Figs. 25, 26, 27, 28, 29, 30 and 31). Therefore during solar peak, the load becomes high as shown in Figs. 23 and 26, and 29 for flexible intervals, non-flexible interval and semi-flexible operation modes respectively. Compared to EM, all techniques of DSM show superior response for utilization of the PV system as shown in Figs. 25 and 28, and 31. During low grid electricity prices, the diesel generator request power is zero and most of power comes from the grid, while at the end of the day due to high grid price and absence of the PV generation, Diesel generator operate to supply the load as shown in Figs. 21, 24 and 27, and Fig. 30.

**Fig. 32:**
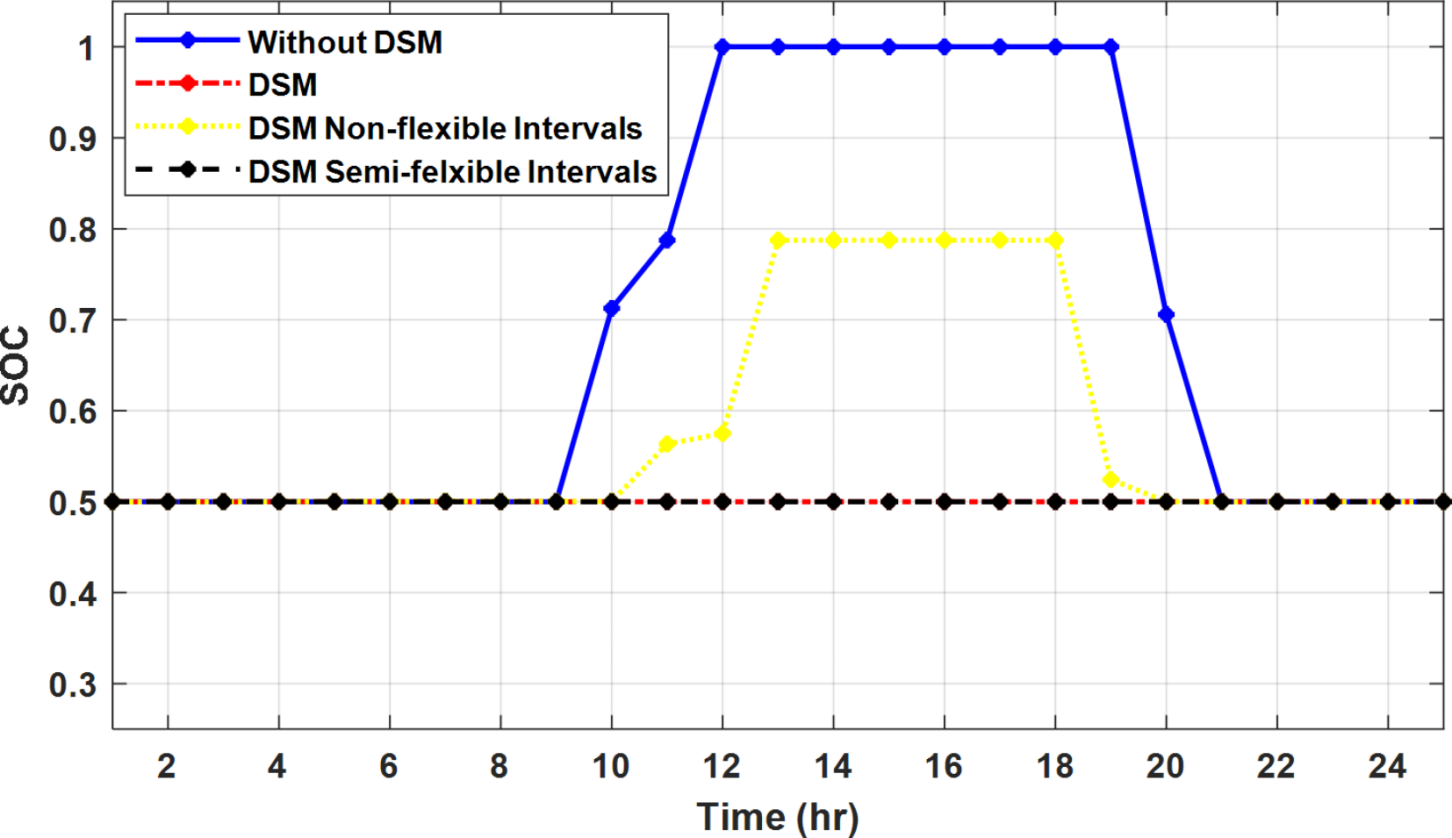
Battery SOC during different operation modes with ToU strategy.

With ToU pricing, the battery charges during surplus of PV generation (such as EM only and EM with DSM incorporating non-flexible intervals). While the battery discharges into the intervals of high grid pricing during the end of the day (18:00 to 20:00) as shown in Fig. 32. As a result, the battery becomes effective during EM with DSM incorporating non-flexible intervals, while still the required energy from battery using EM and DSM is less. The required energy from the battery during DSM with flexible and semi-flexible intervals is zero.

The comparison between different operation modes during ToU strategy is provided in Table 4. With EM only, the NG has a renewable energy curtailment of 18.92%, and the total daily energy cost for the NG is approximately 43.52 $/day. Using EM and DSM with flexible intervals, the generation cost is reduced to 32.54 $/day with cost enhancement of 10.98 $/day, (25.23% cost saving compared to operation without DSM), in addition renewable curtailment is zero. Utilizing DSM with non-flexible intervals, the generation cost increases to 34.32 $ compared to 32.54 $ flexible interval DSM, While the generation cost is reduced by 9.2 $ (21.15%) compared to operation without DSM. This cost reduction is due to using of the most PV generated energy (only 2.17% curtailment compared to 18.92% with EM only). Switching from non-flexible to semi-flexible intervals, the cost improvement becomes 33.77 $/day, with full utilization of the PV system.

**Table 4:** Performance indicators during ToU strategy.

	EM only (without DSM)	EM and DSM (with flexible intervals)	EM and DSM (with non-flexible intervals) (7:00, 12:00, and 18:00)	EM and DSM (with semi-flexible intervals) (7:00, 12:00, and 18:00)
Total generation Cost ($)	43.52 $	32.54 $	34.32 $	33.77 $
Total cost reduction	–	25.23% (10.98 $)	21.15% (9.2 $)	22.42% (9.76 $)
Energy not supplied (%)	0%	0%	0%	0%
Renewable energy curtailment (%)	18.92%	0%	2.17%	0%

### Fixed tariff pricing with utility grid outage

Utility outage scenario is motivated due to managed load shedding programs implanted by utilities to overcome shortage in fossil fuels. The load shedding intervals are announced to customers with sufficient period. To investigate the proposed framework during this operation scenario, the grid tariff is assumed fixed at 0.3 $/kWh and the grid outage occurs at 14:00 and 20:00 and lasts for one hour. During EM, the objective function of the NG management system is to minimize the generation cost and loas curtailment as given by Eq. (2). On the other hand, with DSM, the objective function of the NG management system is given by Eq. (3).

**Fig. 33:**
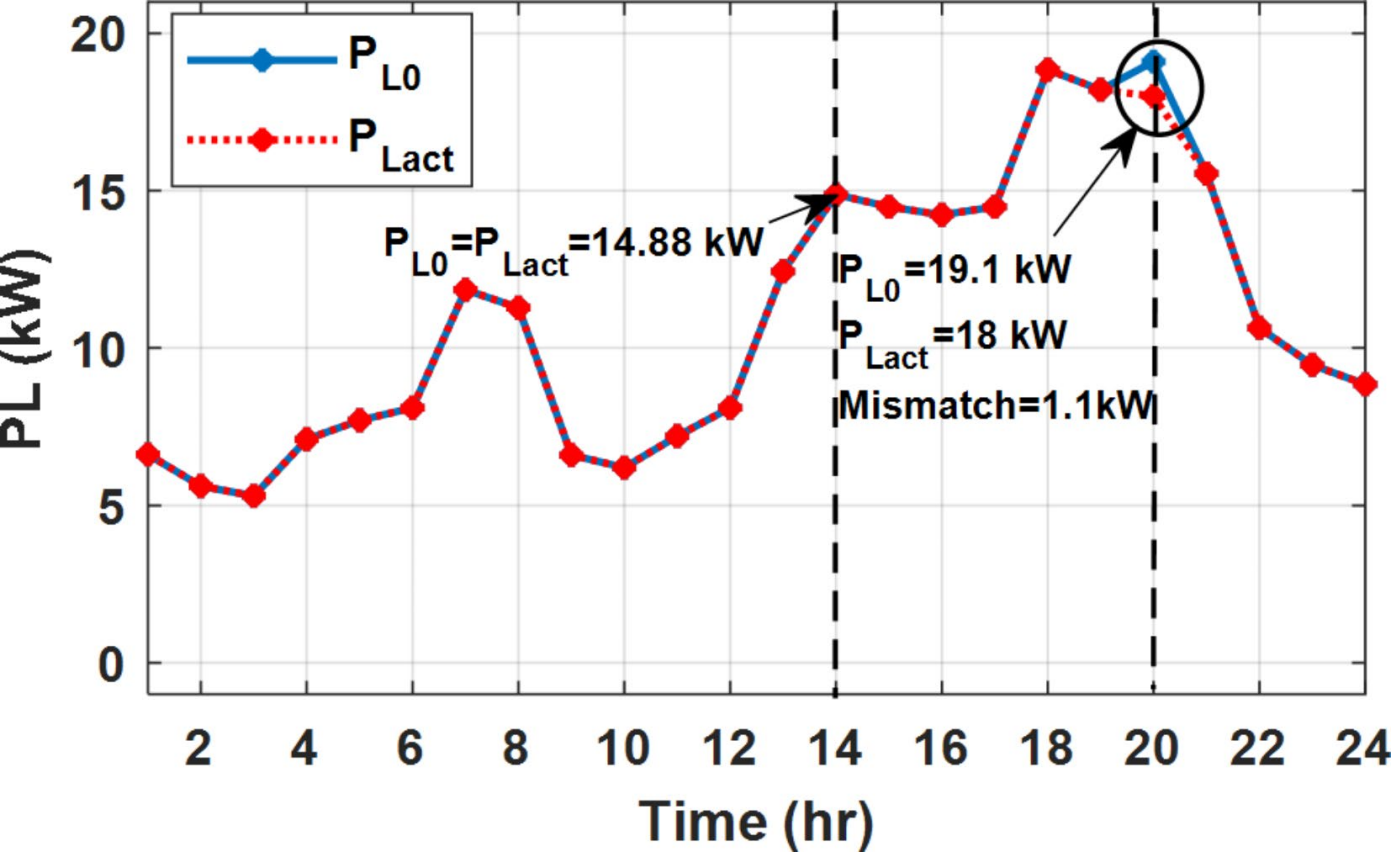
Base and actual load demand during grid outage and FRP without DSM.

**Fig. 34:**
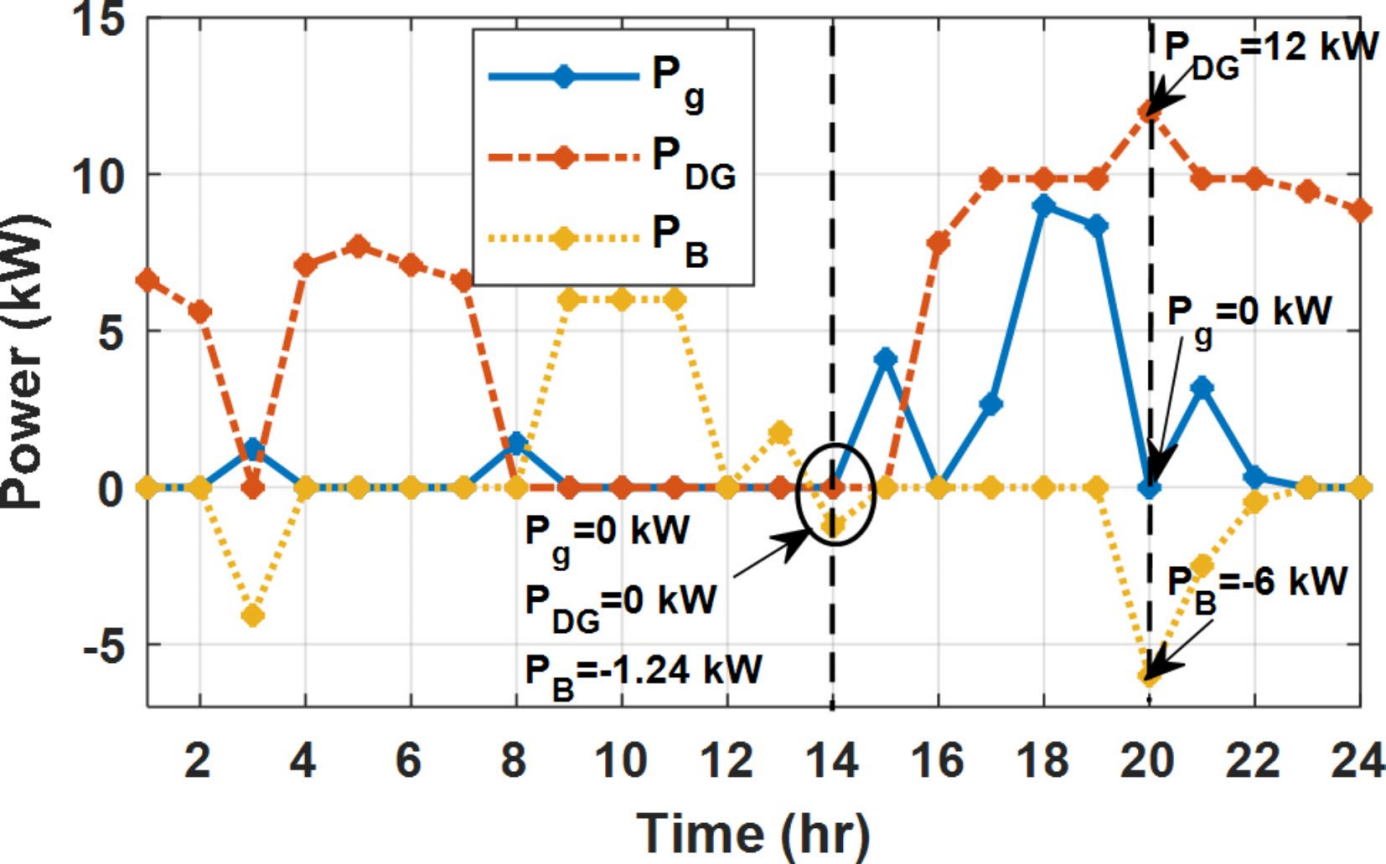
Utility grid, DG, and battery power flow during grid outage and FRP without DSM.

**Fig. 35:**
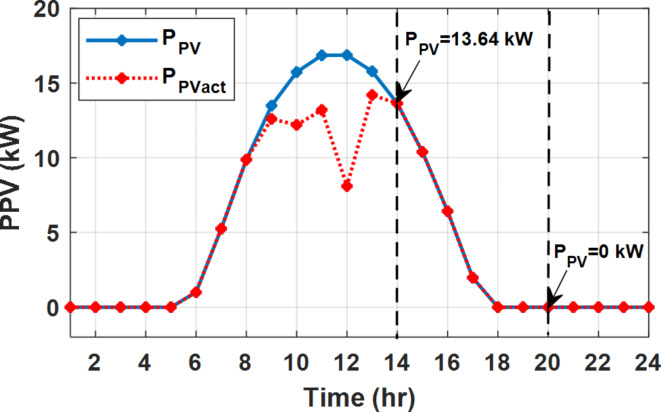
Expected and actual PV power flow during grid outage and FRP without DSM.

**Fig. 36:**
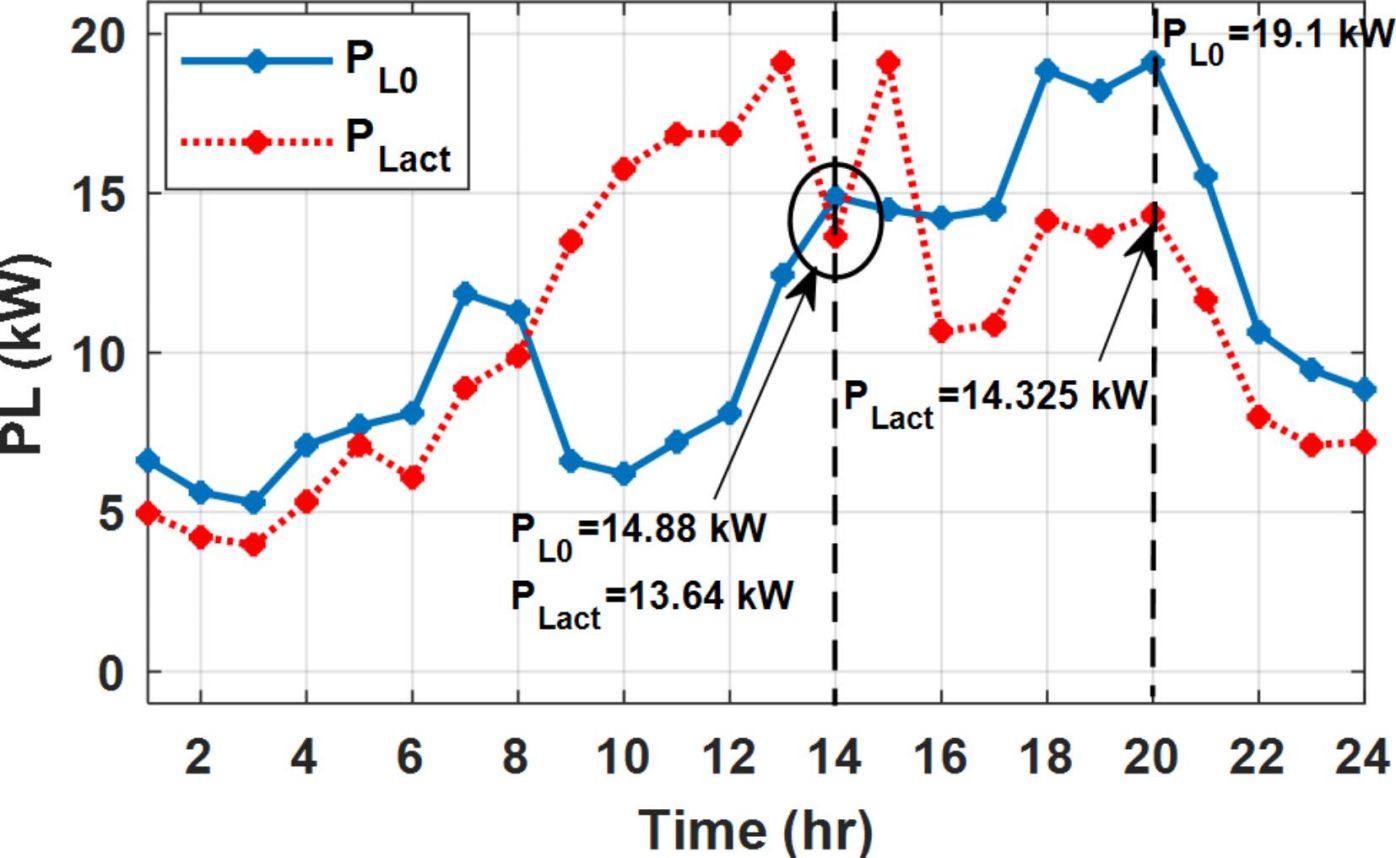
Load demand during grid outage and FRP with/out DSM and flexible intervals.

**Fig. 37:**
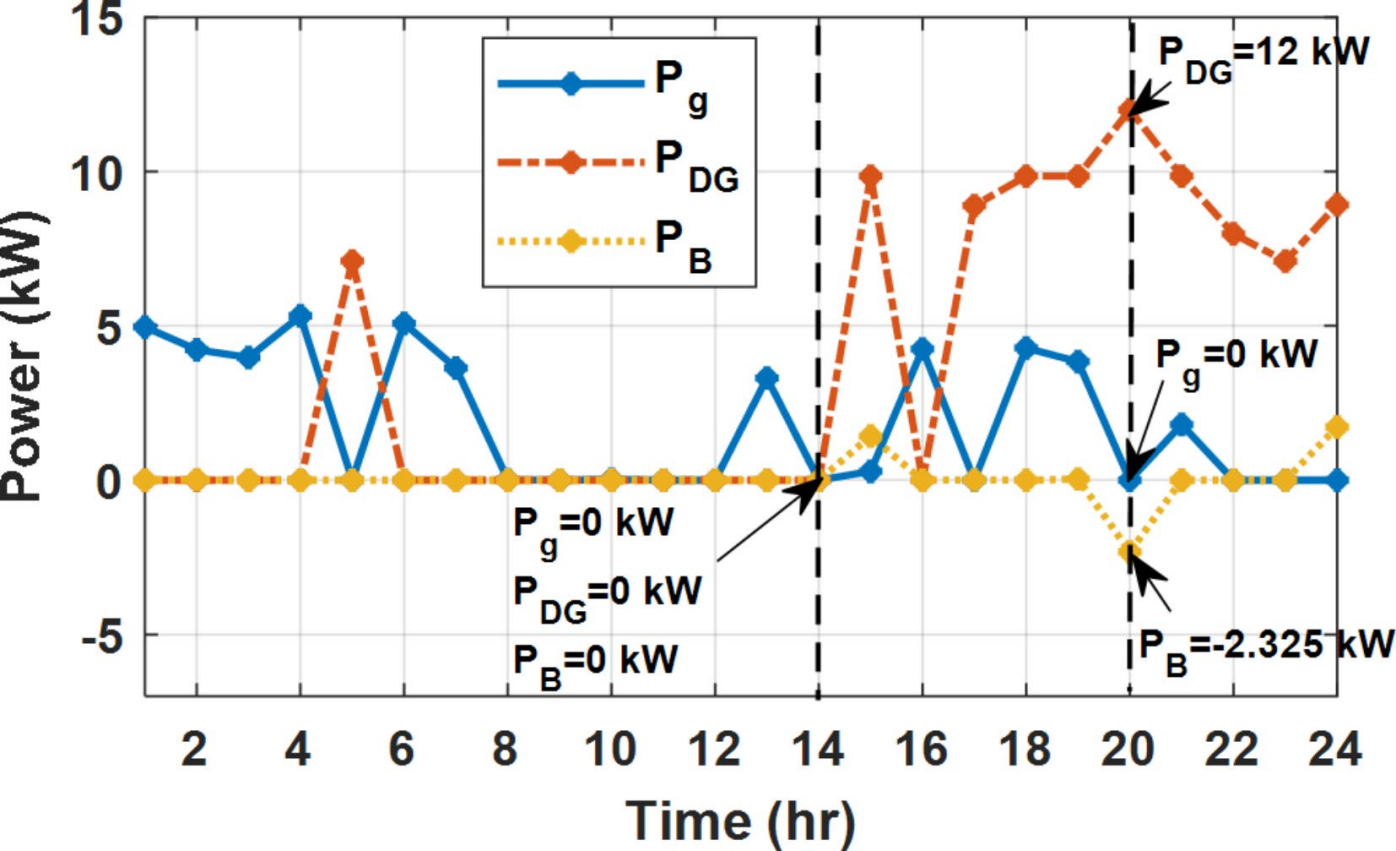
Utility grid, DG, and battery power flow during grid outage and FRP with DSM full flexible.

**Fig. 38:**
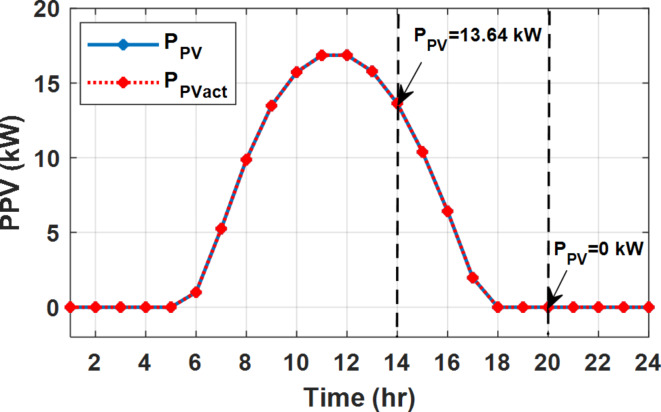
Expected and actual PV Power flow during grid outage and FRP with DSM full flexible.

**Fig. 39:**
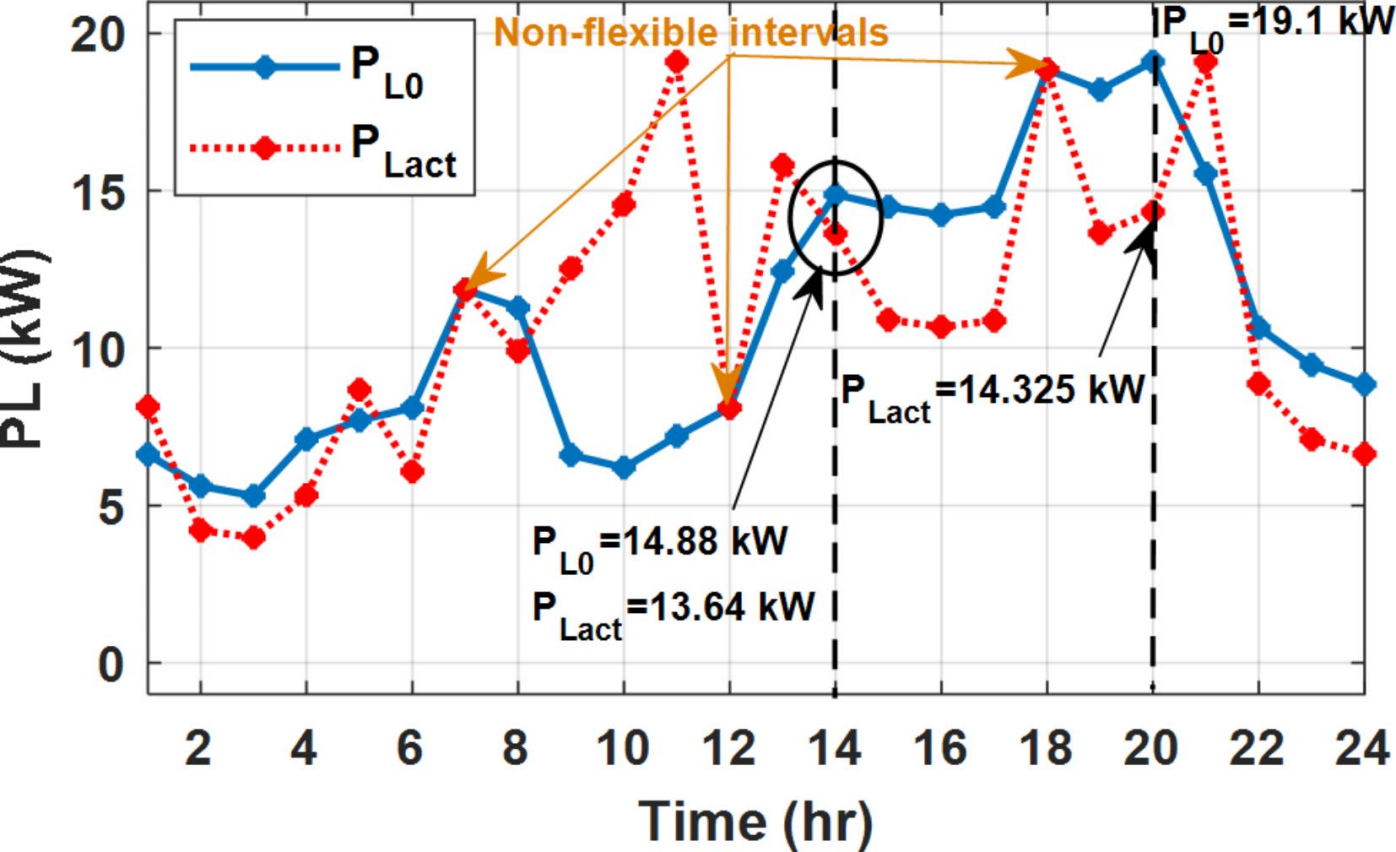
Load demand during grid outage and FRP with/out DSM and non-flexible intervals.

**Fig. 40:**
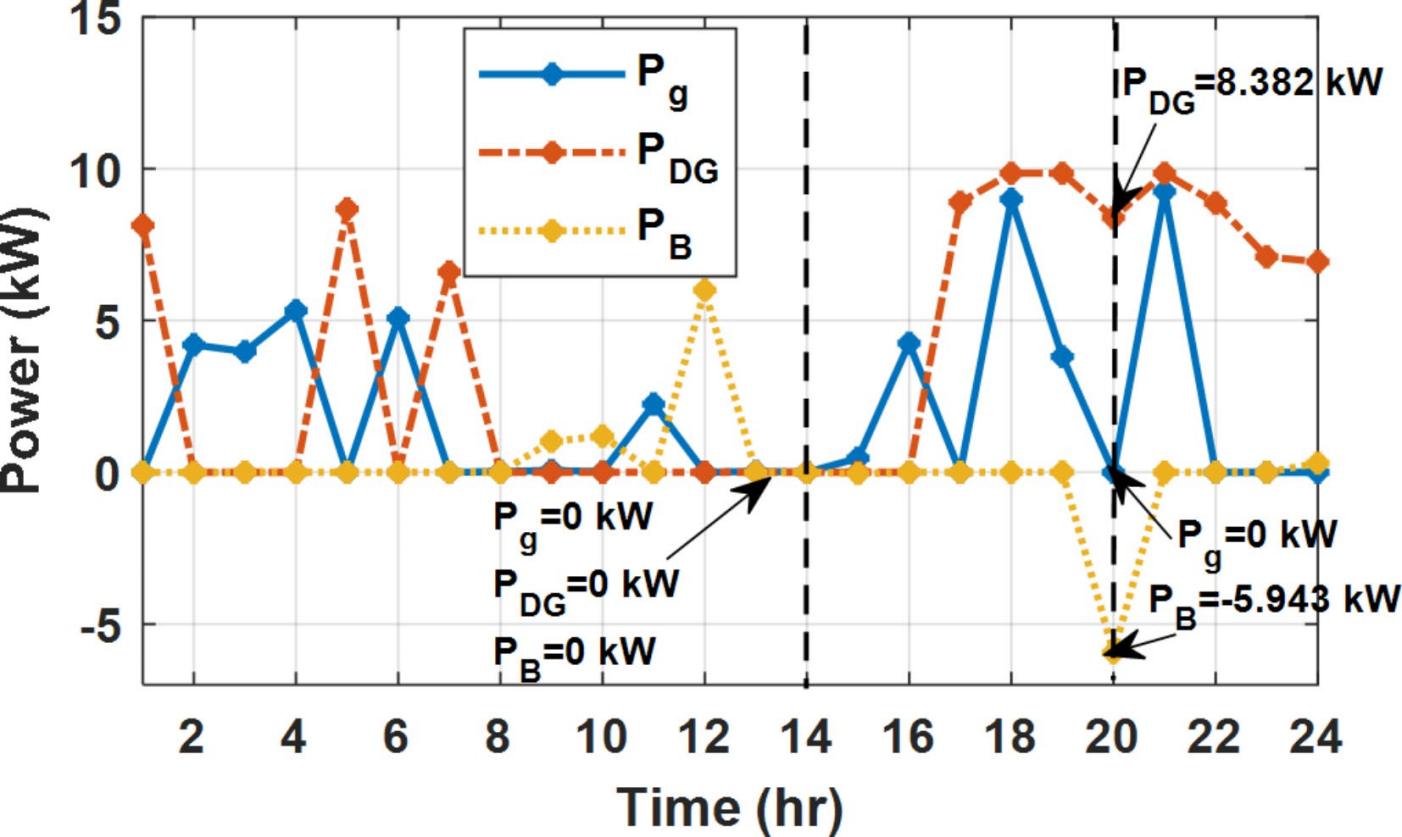
Utility grid, DG, and Battery Power flow during grid outage and FRP with DSM and non-flexible intervals.

**Fig. 41:**
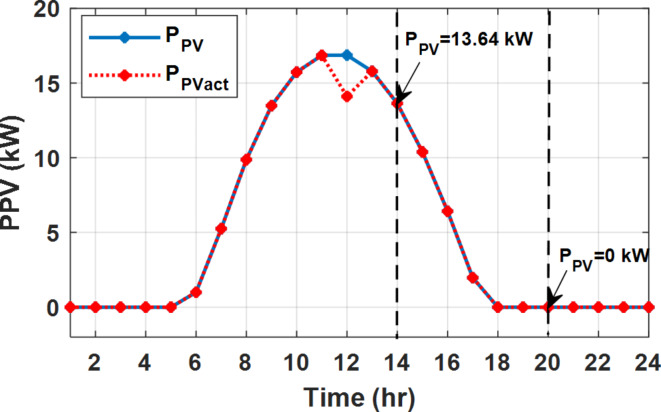
Expected and actual PV Power flow during grid outage and FRP with DSM and non-flexible intervals.

**Fig. 42:**
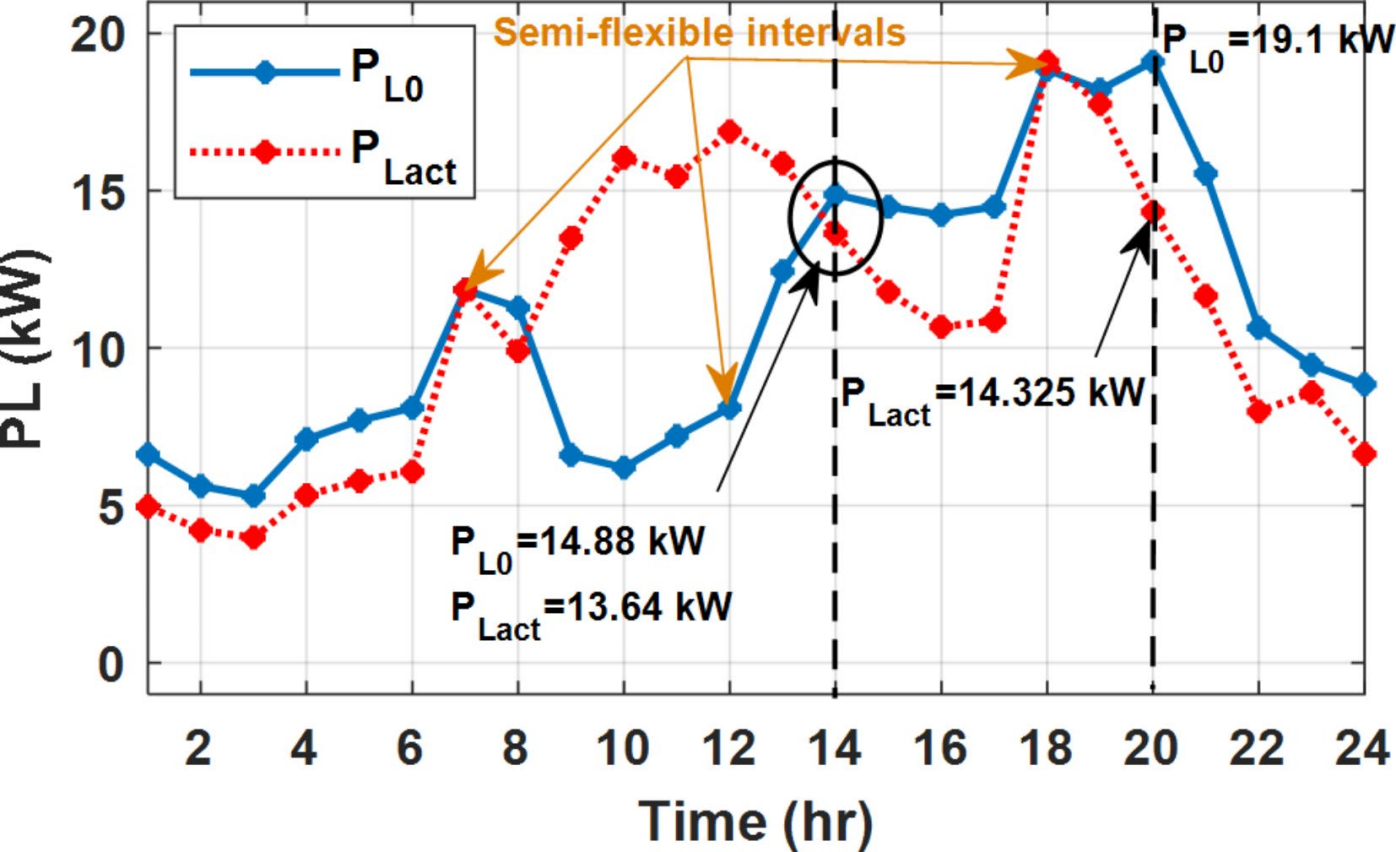
Load demand during grid outage and FRP with/out DSM and semi-flexible intervals.

**Fig. 43:**
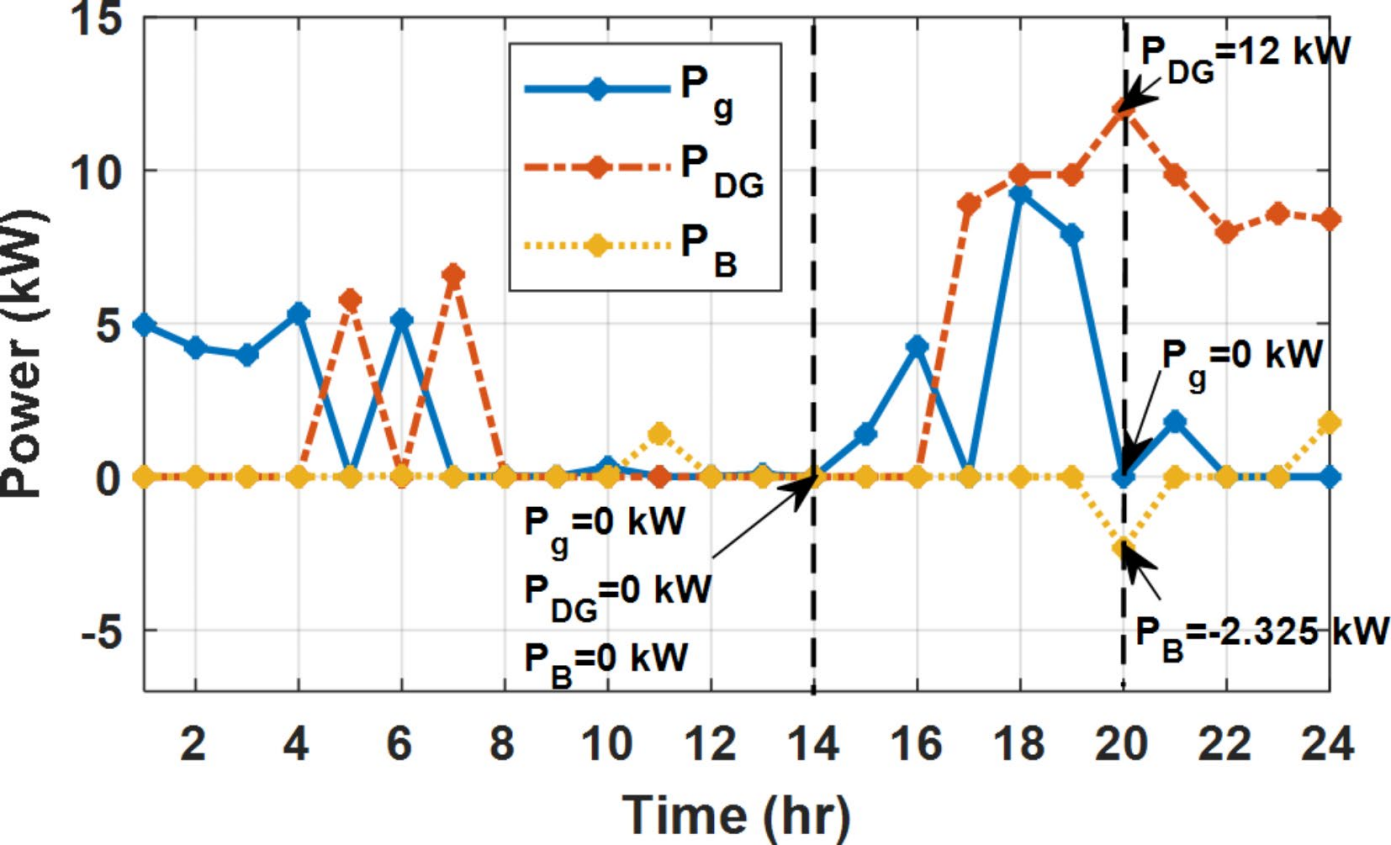
Utility grid, DG, and Battery Power flow during grid outage and FRP with DSM and semi-flexible intervals.

**Fig. 44:**
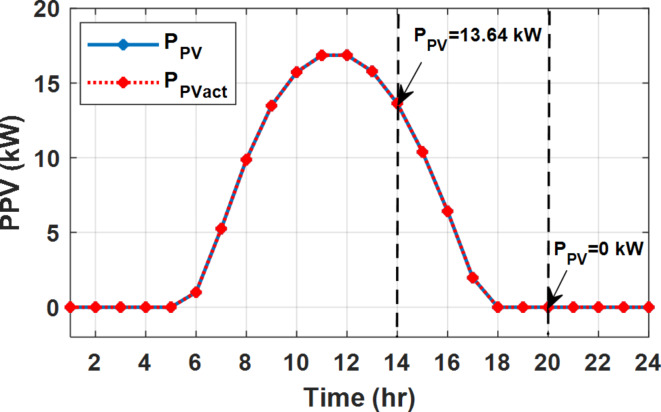
Expected and Actual PV Power flow during grid outage and FRP with DSM and semi-flexible intervals.

During EM only, at grid outage at 14:00, the mismatch between the original load and supplied load is zero as shown in Fig. 33, since the demand load is 14.88 kW which is covered by the Battery (1.24 kW) and the PV (13.64 kW) as shown in Figs. 34, and  35 respectively. While, at 20:00, the customer’s load is 19.1 kW which exceeds the size of the DG (12 kW) and battery (6 kW) (Figs. 36, 37, 38, 39, 40, 41, 42, 43 and 44). Therefore, to minimize the load curtailment, the DG delivers the maximum power (12 kW) and the battery discharge with its maximum rate (6 kW) as shown in as shown in Fig. 34. However, the available power still is less than the required load which causes a demand curtailment by 1.1 kW as shown in Fig. 33. On the other hand, at grid outage at hour 20:00, the load is reduced from 19.1 kW to 14.325 kW as shown in Figs. 36 and 42 respectively. Hence, the load can be supplied by the Diesel (12 kW) and battery (2.325 kW) as shown in Figs. 37, and 43 with flexible and semi-flexible intervals respectively. With non-flexible interval, at grid outage at hour 20:00, the load is reduced from 19.1 kW to 14.325 kW as shown in Fig. 39. Hence, the load is supplied by the Diesel (12 kW) and battery (5.943 kW) as shown in Fig. 40. Consequently, utilizing the DSM, the load curtailment diminishes to zero. The renewable curtailment also comes to zero with flexible and semi-flexible intervals as shown in Figs. 38, and 44 respectively. This restriction on non-flexible intervals reduces the extracted energy from the PV system as shown in Fig. 41.

**Fig. 45:**
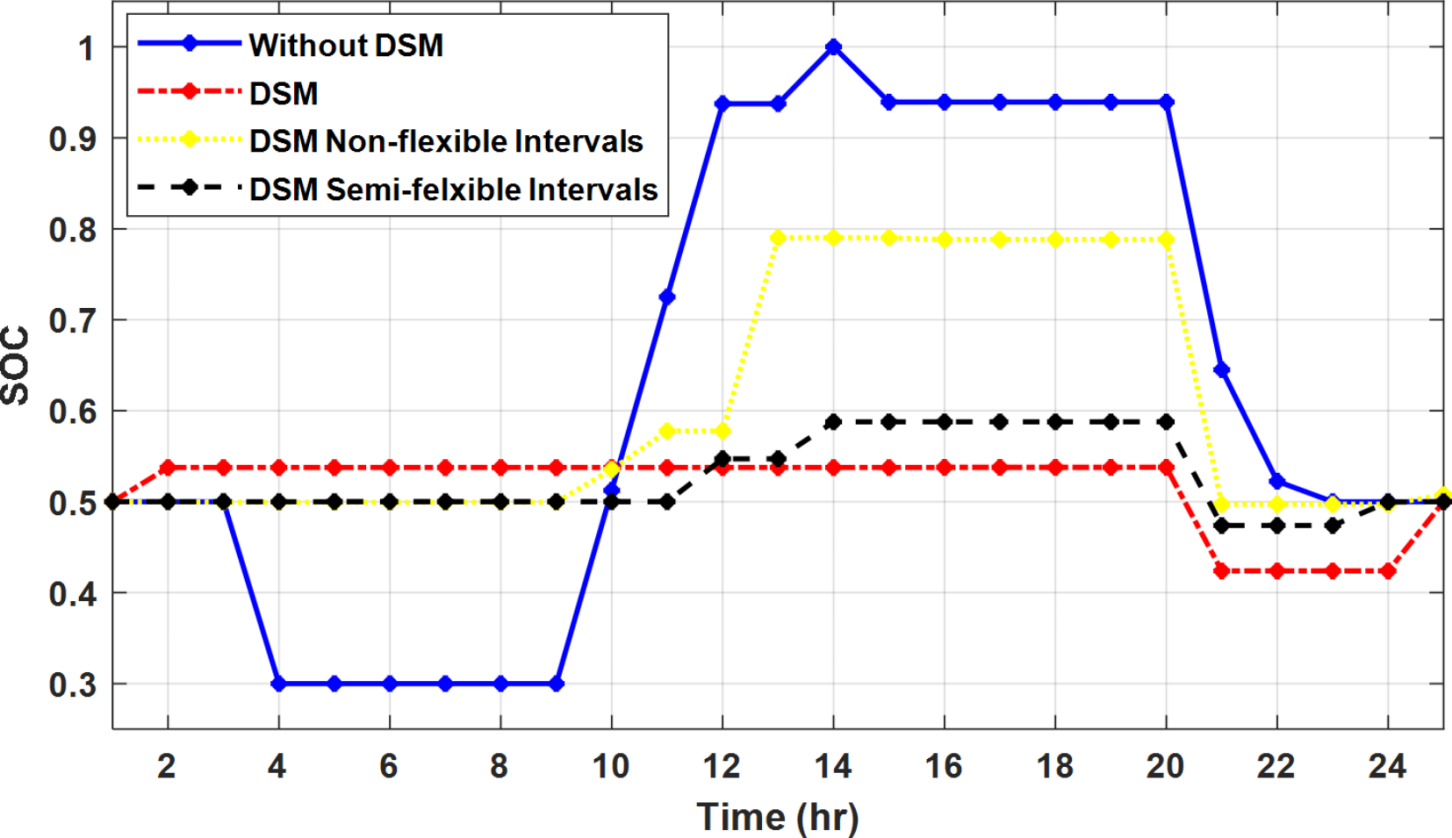
Battery SOC during different operation modes during grid outage and FRP strategy.

During grid outages particularly with EM only, the battery is utilized to compensate for the generation outage because the diesel alone is not sufficient to supply the total load. In addition, the battery is charging during the presence of solar interval and discharge in the other intervals. Therefore, battery is utilized in all operation cases as shown in Fig. 45, while DSM with flexible and semi-flexible intervals, the battery participation is minimum.

Without DSM, the total generation cost is 48.35 $/day, the amount of energy not supplied during grid outage is approximately 1.1 kWh (0.42%) with curtailment in the renewable energy generation about 14.48% as shown in Table 5. However, the generation cost employing DSM with flexible intervals is about 39.58 $/day, with cost enhancement of 8.78 $ (18.15% saving). The benefits extended to reduce the energy not supplied to zero. In addition, renewable energy curtailment is at minimum (zero). With non-flexible intervals, the generation cost decreases to 41.68 $/day which means saving of 6.67 $/day (13.8%) compared to without DSM operation. The percentage of renewable generation curtailment is reduced from 14.48 to 2.17%. Using DSM with semi-flexible intervals, the cost generation falls to 39.74 $/day with cost improvement of 8.61 $/day (17.81%). In addition, the energy not supplied is reduced to zero. Moreover, full utilization of the renewable generation.

**Table 5:** Performance indicators at grid outage with fixed rate.

	EM only (without DSM)	EM and DSM (with flexible intervals)	EM and DSM (with non-flexible intervals) (7:00, 12:00, and 18:00)	EM and DSM (with semi-flexible intervals) (7:00, 12:00, and 18:00)
Total generation Cost ($)	48.35 $	39.58 $	41.68 $	39.74 $
Total Cost reduction	–	18.15% (8.78 $)	13.8% (6.67 $)	17.81% (8.61 $)
Energy not supplied (%)	0.42% (1.1 kWh)	0%	0%	0%
Renewable Energy Curtailment (%)	14.48%	0%	2.17%	0%

### Comparison with published results

**Fig. 46:**
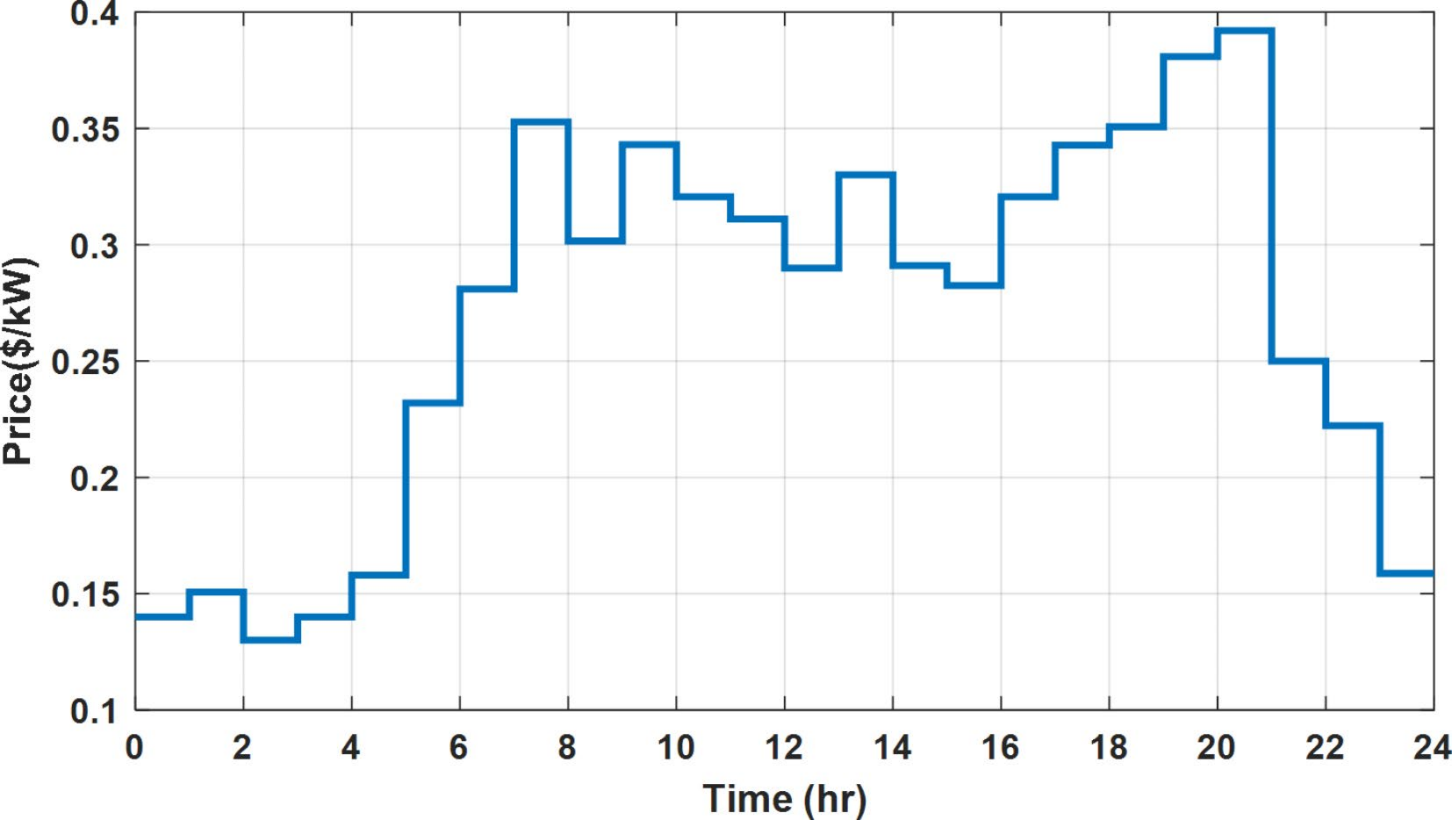
Energy price during RTP strategy^8^.

**Fig. 47:**
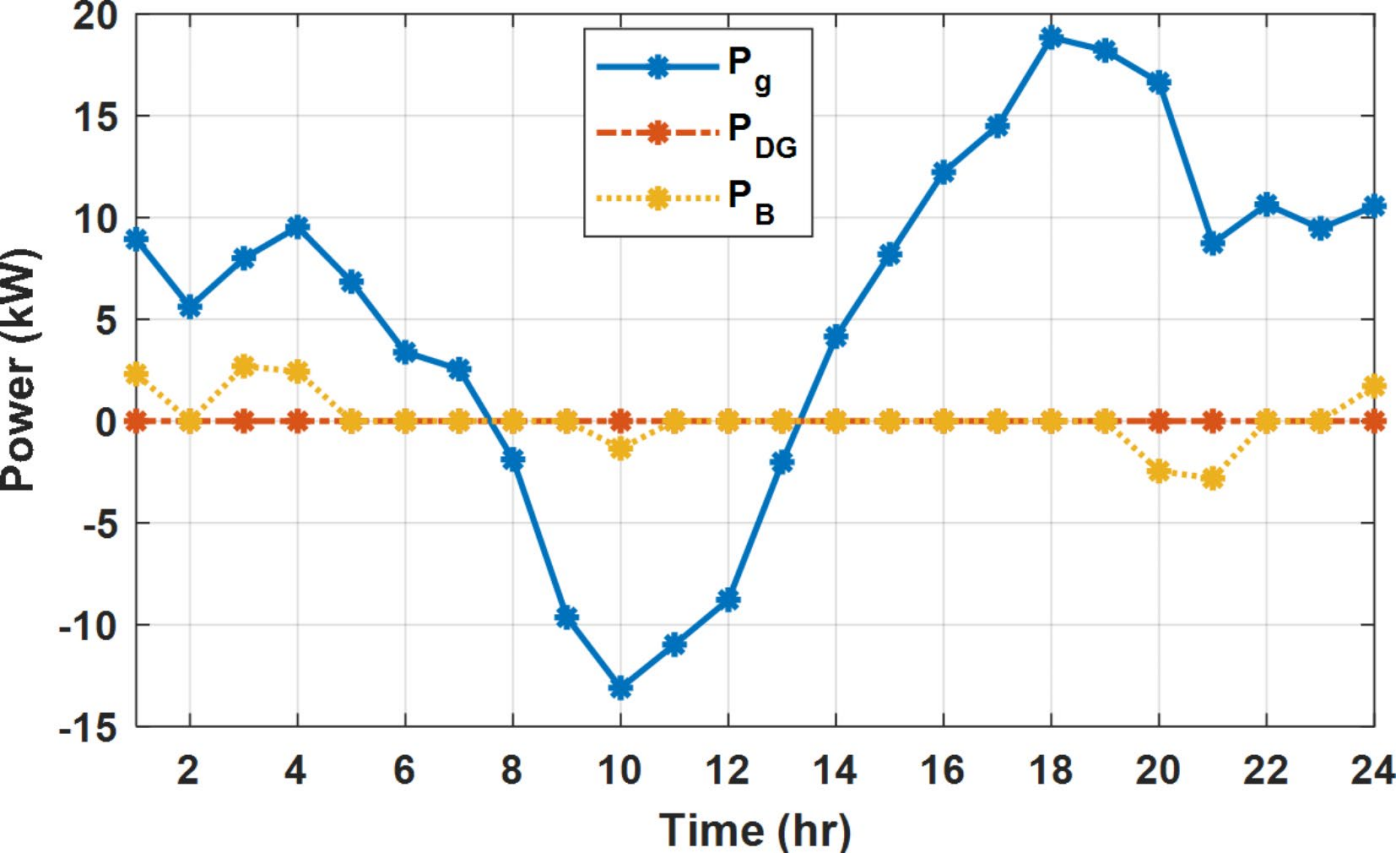
Utility grid, DG, and battery power flow during RTP with EM for NG1 in comparison of published results.

**Fig. 48:**
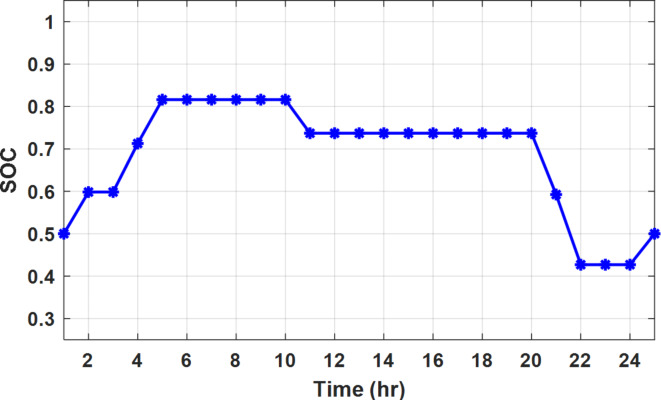
Battery SOC of NG1 in comparison of published results.

To compare the proposed strategy with the published results, the proposed algorithm is applied on a nano-grid typical (is called NG1) to that of ref^8^, since the PV rating is 20 kW, diesel rating is 8 kW, and battery is 20 kWh. Moreover, the grid applies real time pricing (RTP) with bi-directional power flow strategy as shown in Fig. 46. According to ref^8^ with EM only, the daily generation costs are 45.53 $, 45.16 $, and 45.03 $ for particle swarm optimization (PSO), Aquilla optimization (AO), and Improved Aquilla optimization (IAO) techniques respectively. On the other hand, with the proposed methodology, the daily generation cost reaches 33.65 $ which means cost reduction with 11.88 $ (26.1% compared to PSO) as shown in Table 6. This significant reduction is achieved, because the proposed algorithm checks the optimal solution in case of absence of grid, absence of the diesel, and operation of both the grid and diesel. For optimal operation, the diesel unit is turned off throughout the day as shown in Fig. 47 due to the high no load operation cost of the diesel unit 1$/h in ref.^8^. It shall be noted, the grid power is positive in some intervals and negative on the other interval due to the permission of the bi-direction power flows. the battery charges at low pricing periods (23:00 to 05:00) and discharges in during high pricing periods (20:00 to 21:00) as shown in Fig. 48.

**Table 6:** Comparison of the proposed algorithm during energy management with published results:

Algorithm	PSO^8^	AO^8^	IAO^8^	Proposed
Generation cost	45.53	45.16	45.03	33.65
Cost saving relative to PSO	–	0.81	1%	26.1%

## Generation cost and battery participation during different scenarios

**Fig. 49:**
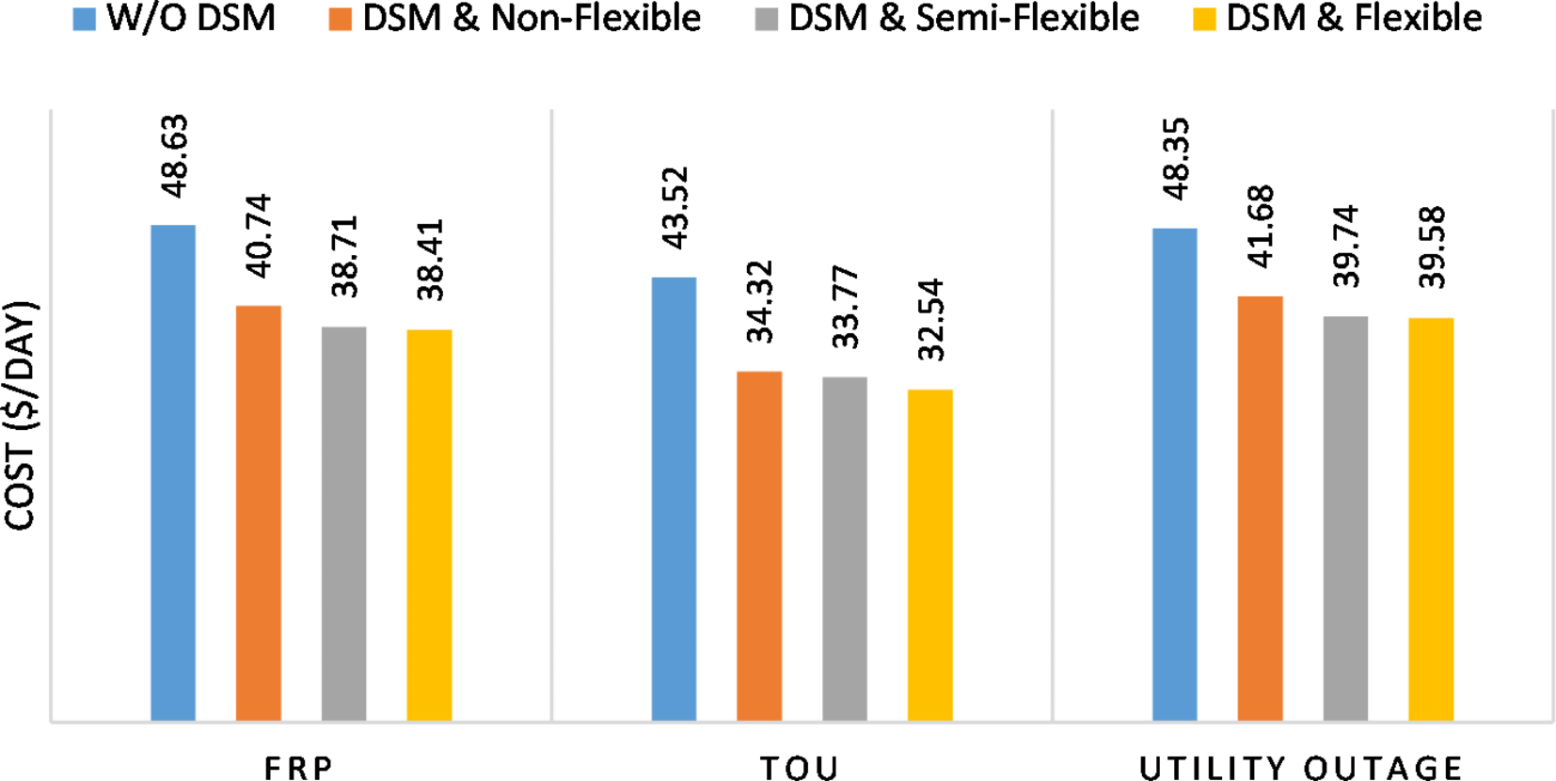
Generation cost per day during different operation modes.

**Fig. 50:**
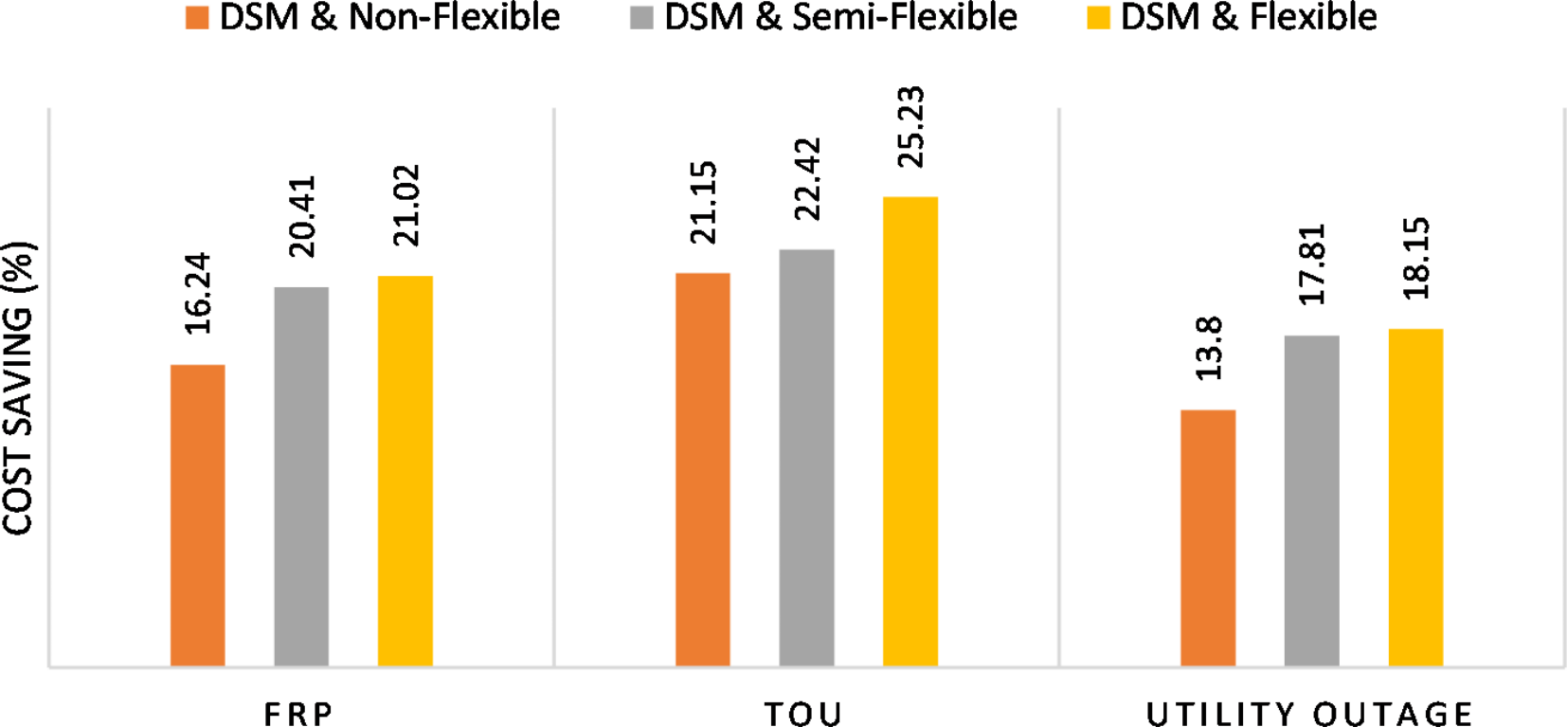
Cost saving percentage during different operation modes.

The total generation cost and total cost saving during different operation modes are shown in Figs. 49 and 50 respectively. For all grid strategies and user preferences, the DSM provides significant cost reduction. For all grid strategies, the DSM with full flexible interval achieves the minimum generation cost. During grid outage (is applied with fixed rate), the generation cost is reduced while it has higher value compared to the FRP with the presence of grid. for the proposed NG, the generation cost saving varies from 13.8 to 25.23% according to the applied user preference and utility applied pricing strategy as shown in Fig. 50.

**Fig. 51:**
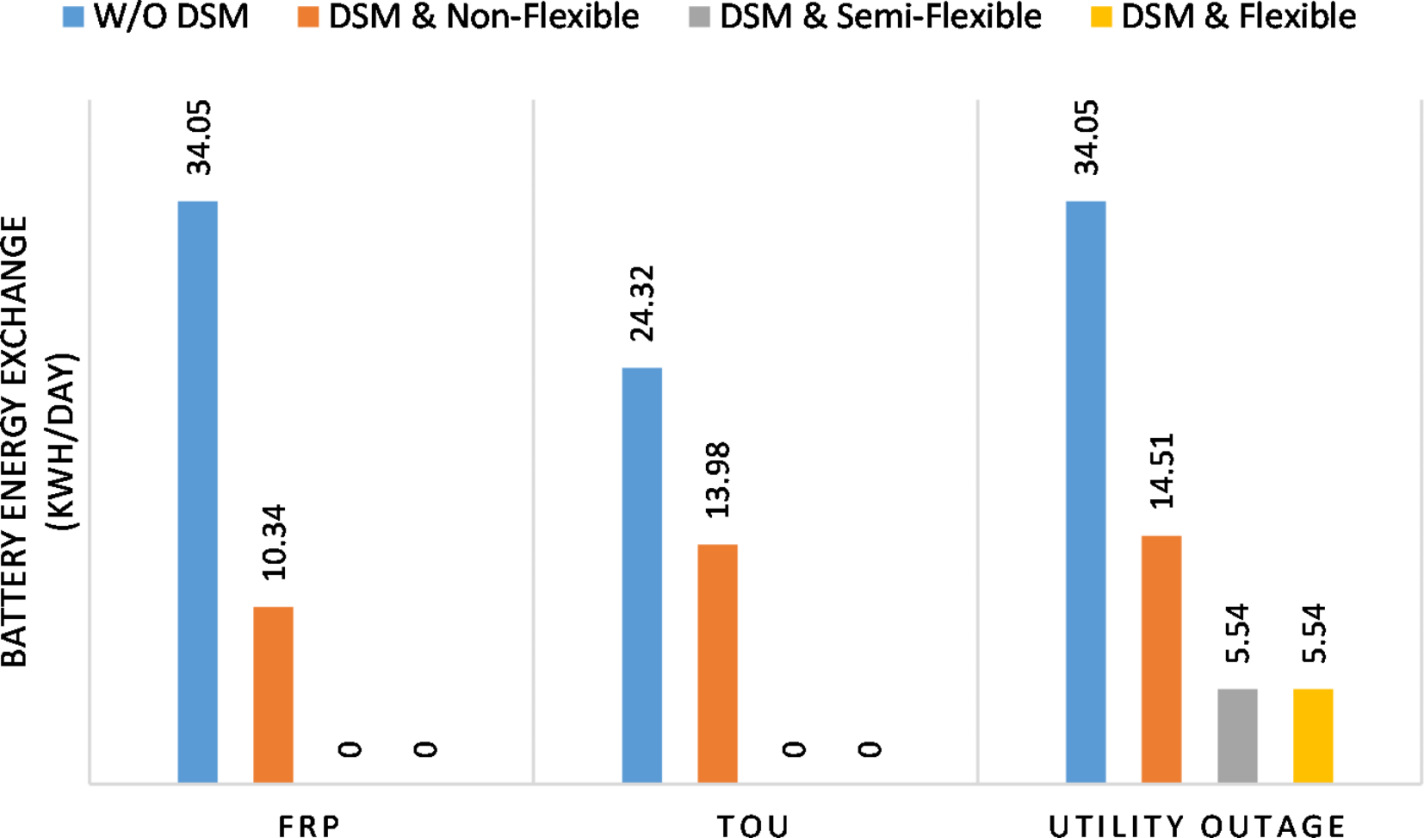
Battery energy exchange (charge plus discharge) during different modes

Figure 51 shows that the total energy storage/release in/from the battery along the day is reduced significantly using DSM. Without DSM, the required energy exchange with battery is high for cost reduction and better utilization of renewable generation. DSM with flexible intervals minimizes the required energy to zero during FRP and ToU strategies, which means the battery can be omitted from the system consequently the system capital cost and required installation space are reduced. With DSM and flexible intervals, the total energy exchange is limited to 5.54 kWh/day during grid outage in the FRP strategy which means reduction about 83.73% compared to without DSM (34.05 kWh/day).

## Evaluation of convergence of the system used algorithms

Due to redness of the Metaheuristic techniques, their stability cannot be ensured from single optimization cycle. Hence, to assist the performance of the proposed hybrid (MFO + Lagrange) compared to the MFO and PSO, the problem is solved 30 independent cycles using 200 population size and number of iterations 2000 per single run. The statistical metrics, including minimum, maximum, mean values and the standard deviation of the obtained results are summarized in Table 7. For all operation scenarios, the MFO + Lagrange succussed to achieve less mean value. 10 out of 12 operation cases, the MFO + Lagrange attains less standard deviation. The obtained results shows that the performance of the MFO + Lagrange is promising compared to MFO or PSO.

**Table 7:** Comparison between convergence results for different algorithms (note: the given values are the fitness function not cost only):

		FRP			ToU			FRP with grid outage		
		Lagrange & MFO	MFO	PSO	Lagrange & MFO	MFO	PSO	Lagrange & MFO	MFO	PSO
EM only (Without DSM)	Max.	48.72	49.07	49.22	43.52	43.56	44.66	50.22	50.38	50.75
	Min.	48.63	48.63	48.63	43.52	43.52	43.52	49.45	49.45	49.45
	Mean	**48.64**	48.75	48.81	**43.52**	43.52	43.69	**49.61**	49.75	49.84
	Standard deviation (σ)	**0.02**	0.10	0.15	**0.00**	0.01	0.30	**0.27**	0.27	0.35
EM and DSM (flexible intervals)	Max.	43.49	42.24	43.77	34.34	35.75	36.52	44.14	44.50	45.86
	Min.	38.41	38.58	38.61	32.54	32.54	32.54	39.58	39.65	39.72
	Mean	**39.96**	40.43	40.49	**33.39**	33.86	33.63	**41.84**	42.01	42.09
	Standard deviation (σ)	1.36	**1.09**	1.39	**0.44**	0.81	0.74	**0.90**	1.10	1.51
EM and DSM (non-flexible intervals) (7:00, 12:00, and 18:00)	Max.	43.67	45.13	45.34	36.53	36.79	36.50	45.91	47.10	48.20
	Min.	40.74	41.09	41.01	34.32	34.32	34.43	41.68	41.77	41.78
	Mean	**41.86**	42.56	42.79	**35.08**	35.21	35.27	**43.17**	43.98	43.61
	Standard deviation (σ)	**0.80**	1.01	0.94	**0.54**	0.55	0.60	**0.92**	1.17	1.29
EM and DSM (semi-flexible intervals)	Max.	43.29	43.46	42.47	36.64	38.47	37.58	44.25	45.60	44.48
	Min.	38.71	38.88	38.92	33.77	33.77	33.77	39.74	40.05	40.27
	Mean	**40.52**	40.97	40.53	**34.59**	35.00	34.76	**42.33**	42.66	42.38
	Standard deviation (σ)	1.29	1.41	**1.11**	**0.67**	1.02	0.74	**1.07**	1.27	1.11

Significant values are in [bold].

## Conclusion and future research

The paper proposed a framework considering EM and DSM opportunities for optimal operation of grid connected NG comprises PV, battery, and diesel generator during fixed rate pricing (FRP), time of use (ToU) pricing, and FRP with grid interruption. Terminologies are presented to describe the relation among the load and intervals denoted as flexible interval, non-flexible interval, and semi-flexible interval. Since in flexible interval, the base load is controllable, and the interval can receive additional loads. Non-flexible interval is the interval which the load cannot fluctuate from its base value and that interval cannot receive additional loads. Semi-flexible interval, the load is not controlled (cannot decreased or shifted) while the interval can receive more loads from other intervals. Four customers preferences are investigated defined as EM without DSM, EM and DSM with all flexible intervals, EM and DSM with non-flexible intervals, and EM with DSM and semi-flexible intervals. The optimization problem is solved using hybrid Lagrange and Moth-flame Optimization (MFO) algorithm which exhibits promising performance compared to MFO or PSO.

Considering both EM and DSM simultaneously in the proposed framework provides a significant reduction in the cost. DSM with flexible intervals reduces the generation cost by 21.02%, 25.23%, and 18.15% for FRP, ToU, and FRP with grid outage scenarios respectively. While DSM with semi-flexible intervals reduces the generation cost by 20.41%, 22.42%, and 17.81%, respectively. Eventually, DSM with non-flexible intervals the cost is reduced by 16.24%, 21.15%, and 13.8%, respectively for non-flexible intervals respectively. Hence, the DSM with flexible intervals provides better opportunity to optimize the generation cost compared to other operation scenarios. This cost reduction is associated with better utilization of renewable energy generation (0% renewable curtailment in flexible and semi-flexible intervals).

During ToU pricing strategy, the proposed framework utilizes the variation of the electricity price to shift the loads from the higher price intervals to the lower pricing intervals. In addition, the proposed framework charges the battery during lower pricing interval and discharges the battery into the load during higher pricing interval. These both actions performed simultaneously which produces much remarkable cost reduction.

Without DSM, at grid outage, the power balance constraint is not satisfied when the available generation is less than the load. Therefore, part of the load shall be curtailed by the amount of power mismatch to make the problem feasible. However, this generation shortage depends on the interval of interruption. To address this challenge, the proposed framework considers the amount of power not supplied into the objective function. It is notable that with DSM, When the generated power is less than the load during grid outage, the proposed framework optimally shifts the load to other internals with sufficient generation for minimization of the energy cost and the energy not supplied.

The DSM manages the load to provide better utilization of available generation resources with less amount of energy storage along the day and less amount of peak energy storage. The peak of energy reduction leads to a reduction of the required battery system capacity, consequently a reduction in the system capital cost and the required space. In some cases, such as FRP and ToU, the DSM optimizes the system without using energy storage which enables customers to avoid the battery during the erection of the system.

Due to importance and increasing the integration of smart grids (Micro-grid, and Nano-grid) based renewable energy resources with energy management and demand side management, this research can be extended in future work to include additional objectives such as emission minimization, also studying the system for real time operation considering uncertainty of the meteorologist data (solar irradiance, ambient temperature), the uncertainty of the load, and consumer equipment characteristics. Moreover, the work can be extended to include larger systems such as cluster of nano-grids.

## Data Availability

All data generated or analyzed during this study are included in this published article.

## List of symbols

$$P_{PV}$$: Solar output power from PV. (kW)
$$P_{STC}$$: Power retrieved from the PV panel at the standard testing conditions. (W/m^2^)
$$I_{s}$$: Solar irradiance (W/m^2^)
$$\rho_{PV}$$: Temperature power coefficient of the module
$$T_{c}$$: Module temperature (°C)
$$T_{a}$$: Ambient temperature (°C)
$$T_{n}$$: Nominal module temperature (°C)
$$C_{g} \left( t \right)$$: Cost of energy supplied from the grid ($)
$$C_{dg} \left( t \right)$$: Cost of energy supplied from the diesel ($)
$$C_{B} \left( t \right)$$: Cost of energy supplied from the battery ($)
*a*, *b* and *c*: Generator fuel cost coefficients from manufacture data. ($/kW^2^, $/kW, and $ respectively)
$$\rho_{g} \left( t \right)$$: Grid tariff ($/kW)
$$P_{g} \left( t \right)$$: Supplied power from the grid at time t. (kW)
$$\rho_{B}$$: Battery usage cost coefficient ($${\text{\$ }}/kWh)$$
$$P_{B} \left( {\text{t}} \right)$$: Battery power during time t. (kW)
$$P_{L} \left( {\text{t}} \right)$$: Load power at time t. (kW)
$$P_{dg} \left( t \right)$$: Diesel generator output power at time t. (kW)
$$P_{PV\_act} \left( t \right)$$: Actual PV power at time t. (kW)
$$\eta_{ch}$$: Charging efficiency of the batteries (%)
$$\rho_{PV}$$: Temperature coefficient of the module power (%/°C)
$$W_{n}$$: Battery Energy capacity (kWh)
$$P_{meter}$$: Maximum metering power. (kW)
$$P_{Lo} \left( t \right)$$: Load Power after implementing DSM. (kW)
$$P_{L\_act} \left( t \right)$$: Actual load Power (kW)
$$P_{L\_min} \left( t \right)$$: Minimum load Power (kW)
$$P_{L\_max} \left( t \right)$$: Maximum load Power (kW)
n: Initial number of flames (or number of Moths)
*d*: Problem variables (dimensions).
F, M: Flame and Moths matrix respectively
OM: Fitness vector corresponding to the Moths matrix M or the flame matrix *F*
k: Random number k ∈ [-1,1].
*Di*: Distance from moth *i* and flame *j*.
T: Total number of iterations
*l*: Current iteration number
*ui* & *l*i: Upper and lower boundaries of moth *i*
$$E_{N.S}$$: Energy not Supplied (%)
$$REC$$: Renewable Energy Curtailment (%)
$$\eta_{dis}$$: Discharging efficiency of the batteries (%)

## Abbreviations

DSM: Demand side management
NG: Nano-grid
FRP: Fixed rate pricing
ToU: Time of use
RESs: Renewable energy sources
EM: Energy management
DG: Diesel generator
PV: Photovoltaic panels
BES: Battery energy storage system
PSO: Particle swarm optimization technique
MFO: Moth-flame optimization technique
SOC: The state of charge of the batteries
